# A gold speciation that adds a second layer to synergistic gold-copper toxicity in *Cupriavidus metallidurans*

**DOI:** 10.1128/aem.00146-24

**Published:** 2024-04-01

**Authors:** Niklas Hirth, Nicole Wiesemann, Stephanie Krüger, Michelle-Sophie Gerlach, Kilian Preußner, Diana Galea, Martin Herzberg, Cornelia Große, Dietrich H. Nies

**Affiliations:** 1 Molecular Microbiology, Institute for Biology/Microbiology, Martin Luther University Halle-Wittenberg, Halle (Saale), Germany; 2 Microscopy Unit, Biocenter, Martin Luther University Halle Wittenberg, Wittenberg, Germany; 3 Department of Analytical Chemistry, Helmholtz Centre for Environmental Research – UFZ, Leipzig, Germany; University of Michigan, Ann Arbor, Michigan, USA

**Keywords:** copper, gold, *Cupriavidus metallidurans*

## Abstract

The metal-resistant bacterium *Cupriavidus metallidurans* occurs in metal-rich environments. In auriferous soils, the bacterium is challenged by a mixture of copper ions and gold complexes, which exert synergistic toxicity. The previously used, self-made Au(III) solution caused a synergistic toxicity of copper and gold that was based on the inhibition of the CupA-mediated efflux of cytoplasmic Cu(I) by Au(I) in this cellular compartment. In this publication, the response of the bacterium to gold and copper was investigated by using a commercially available Au(III) solution instead of the self-made solution. The new solution was five times more toxic than the previously used one. Increased toxicity was accompanied by greater accumulation of gold atoms by the cells. The contribution of copper resistance determinants to the commercially available Au(III) solution and synergistic gold-copper toxicity was studied using single- and multiple-deletion mutants. The commercially available Au(III) solution inhibited periplasmic Cu(I) homeostasis, which is required for the allocation of copper ions to copper-dependent proteins in this compartment. The presence of the gene for the periplasmic Cu(I) and Au(I) oxidase, CopA, decreased the cellular copper and gold content. Transcriptional reporter gene fusions showed that up-regulation of *gig*, encoding a minor contributor to copper resistance, was strictly glutathione dependent. Glutathione was also required to resist synergistic gold-copper toxicity. The new data indicated a second layer of synergistic copper-gold toxicity caused by the commercial Au(III) solution, inhibition of the periplasmic copper homeostasis in addition to the cytoplasmic one.

**IMPORTANCE:**

When living in auriferous soils, *Cupriavidus metallidurans* is not only confronted with synergistic toxicity of copper ions and gold complexes but also by different gold species. A previously used gold solution made by using *aqua regia* resulted in the formation of periplasmic gold nanoparticles, and the cells were protected against gold toxicity by the periplasmic Cu(I) and Au(I) oxidase CopA. To understand the role of different gold species in the environment, another Au(III) solution was commercially acquired. This compound was more toxic due to a higher accumulation of gold atoms by the cells and inhibition of periplasmic Cu(I) homeostasis. Thus, the geo-biochemical conditions might influence Au(III) speciation. The resulting Au(III) species may subsequently interact in different ways with *C. metallidurans* and its copper homeostasis system in the cytoplasm and periplasm. This study reveals that the geochemical conditions may decide whether bacteria are able to form gold nanoparticles or not.

## INTRODUCTION

**Fig 1:**
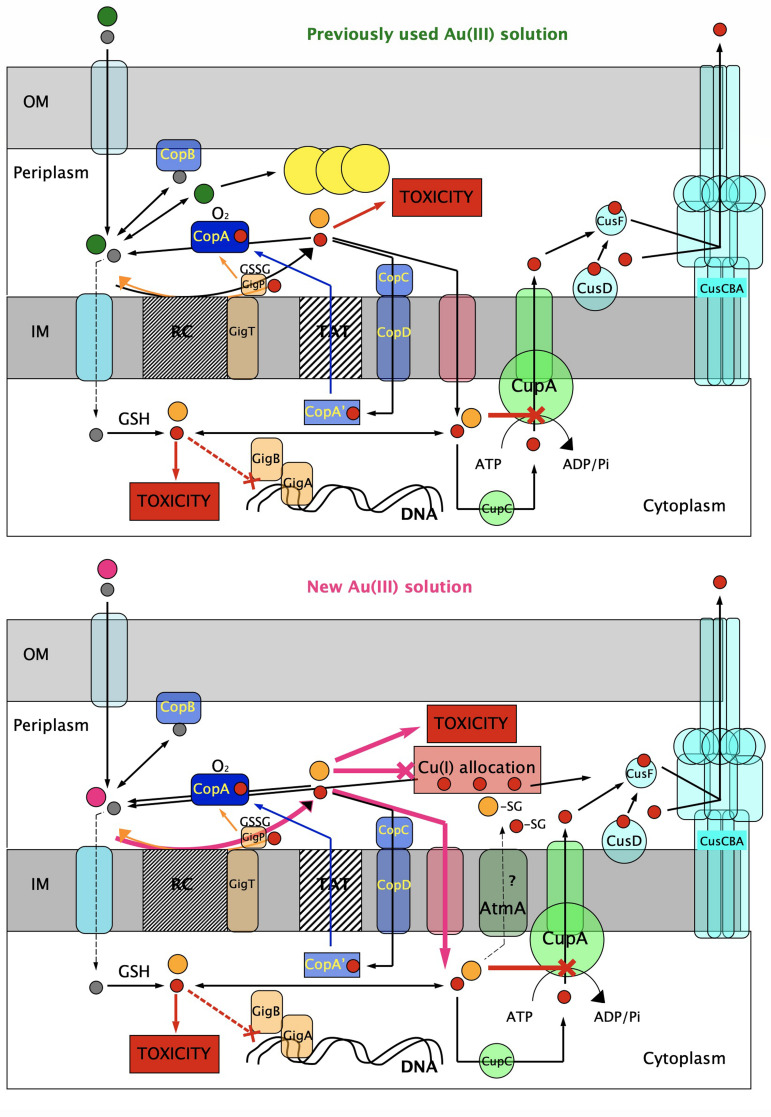
Different gold species cause different effects on *C. metallidurans*. The figure summarizes the effects of the previously used, self-made (top) and new, commercially available Au(III) solution as an interplay with cellular copper homeostasis. Cu(II) (gray circles), Cu(I) (red circles), previously used Au(III) complexes (green circles, possibly Au(III)Cl_3_OH^−^), new Au solution (purple circles, possibly Au(III)Cl_4_^−^), Au(I) species (orange circles), periplasmic Au nanoparticles (big Au circles), details in the text and references (7, 9, 10).

The betaproteobacterium *Cupriavidus metallidurans* survives in environments rich in transition metals (1–3). The necessary metal resistance determinants are located on the bacterial chromosome, a chromid, and two plasmids (4). *C. metallidurans* also occurs in biofilms of biologically formed Au (5). In auriferous soils, Au complexes are rapidly accumulated within the cell but are later precipitated as Au nanoparticles in the periplasm (6). Defense systems against oxidative stress and copper resistance systems are up-regulated upon contact with Au complexes (7). Auriferous soils usually contain an elevated Cu content; consequently, Cu ions and Au complexes exert synergistic Au-Cu toxicity (Fig. 1) because cytoplasmic Au compounds inhibit the P_IB1_-type ATPase CupA, which is responsible for the removal of surplus cytoplasmic Cu(I) ions (8). To counteract this synergistic toxic effect, *C. metallidurans* induces the *cop* determinant. The periplasmic Cu(I)/Au(I) oxidase CopA oxidizes both compounds back to Au(III) and Cu(II), which are less toxic than the respective monovalent cations. Au(III) complexes can be subsequently reduced to metallic Au(0) in the periplasm, leading to direct formation of Au nanoparticles without using the toxic Au(I) state as an intermediate (9) (Fig. 1, top).

From the periplasm, Au ions are imported by unspecific import systems into the cytoplasm (7) and bind to the regulator CupR of the gene *cupA*, which encodes the P_IB1_-type copper-exporting ATPase (11), and the copper chaperone, CupC (12). In the cytoplasm, Au ions should interact with glutathione, reminiscent to the interaction of Cu ions with this tripeptide (13). Moreover, the *gig* determinant (gold-induced genes) may also contribute to Au-Cu resistance; however, *cup* or *gig* is not required to confer resistance to Au (7).

In *C. metallidurans*, the *cup, cop, cus,* and *gig* determinants and GSH cooperate to mediate the full level of copper resistance in the plasmid-free mutant strain AE104 (10) (Fig. 1). Resistance to the synergistic Au-Cu toxicity is not different in strain AE104 compared to its parent strain, CH34 (8, 9). When AE104 cells are pre-incubated in the presence of Cu ions, they acquire increased resistance to the synergistic toxicity of Au(III) and Cu(II). This increase in resistance depends on the Cop system, which is centered around the periplasmic Cu(I) and Au(I) oxidase CopA (9). Copper resistance results from an interplay between many resistance systems, and all these systems are also up-regulated by Au complexes so that these resistance systems may also contribute to Au resistance.

At this point, we noted that commercially available Au(III) chloride solutions (Sigma-Aldrich) had different effects on *C. metallidurans* AE104 cells than the self-made Au(III) chloride solution used previously, which had been prepared by dissolving metallic Au in *aqua regia* to prepare HAuCl_4_ × 3 H_2_O, then dissolving this substance in deionized water and adjusting the concentration to 50 mM (6). These two Au(III) solutions appear to have different chemical properties with respect to the geobiological cycling of Au in nature (14). This prompted us to investigate in more detail the effect of commercially available Au(III) chloride solutions on *C. metallidurans*. We found that these solutions were five times more toxic than the self-made ones, did not lead to the formation of Au nanoparticles in the periplasm, and interacted with the copper resistance determinants of *C. metallidurans*. It added a second layer to the synergistic Au-Cu toxicity in this bacterium: inhibition of the periplasmic in addition to the cytoplasmic copper homeostasis, which was also caused by the self-made solution. These analyses have revealed how two different Au(III) chemistries can differentially influence the copper homeostasis of *C. metallidurans*.

## RESULTS

### The speciation of the gold complexes determines their toxicity

The self-made Au(III) solution had an IC_50_ value on *C. metallidurans* AE104 cells of 28 µM (Table 1). While deletion of the *cop* determinant for the periplasmic Au(I) oxidase, CopA, decreased gold resistance (9), the *cus* and the *gig* determinants did not decrease resistance to self-made Au(III) solutions (Table 1). Deletion of the *cup, cus,* or *gig* determinants individually also had only a small effect on the synergistic Au-Cu toxicity or induction of resistance to this condition by pre-incubation in the presence of elevated Cu concentrations (Fig. S1).

**TABLE 1:** Gold resistance of *C. metallidurans* deletion mutants^*a*^

Bacterial strain	IC_50_	*Q*; *D*
	Previous Au(III) solution	
AE104	28.3 ± 2.6	1.00; 0.00
*Δcus*	24.6 ± 3.1	0.87; 0.67
*Δgig*	25.2 ± 1.4	0.89; 0.78
	New Au(III) solution	
AE104	5.74 ± 0.63	1.00; 0.00
*ΔgshA*	4.81 ± 0.49	0.84; 0.83
*Δcup*	5.44 ± 0.64	0.95; 0.24
*Δgig*	5.39 ± 1.03	0.94; 0.21
*Δcop*	5.27 ± 0.76	0.92; 0.34
*Δcus*	**8.96 ± 1.77**	**1.56; 1.34**
*Δcop Δcus*	5.96 ± 0.87	1.04; 0.15
*Δcup Δcus*	4.67 ± 0.74	0.81; 0.78
*Δcop Δcup*	4.99 ± 0.74	0.87; 0.55
*Δcup Δgig*	6.67 ± 0.53	1.16; 0.80
*Δgig Δcus*	**11.60 ± 1.30**	**2.02; 3.04**
*Δcop Δgig*	**11.40 ± 1.80**	**1.99; 2.33**
*Δcop Δcus Δgig*	4.84 ± 0.59	0.84; 0.74
*Δcup Δcus Δgig*	5.50 ± 0.70	0.96; 0.18
*Δcop Δcup Δgig*	**12.30 ± 0.91**	**2.14; 4.26**
*Δcop Δcup Δcus*	**10.60 ± 1.78**	**1.85; 2.02**
*Δcop Δcup Δcus Δgig*	**11.20 ± 1.10**	**1.95; 3.16**

^*a*^ The IC_50_ values were calculated from dose-response experiments. Comparison was between a mutant and AE104 parent in the presence of the previously used or new gold solution. *Q* is the ratio of the IC_50_ of mutant strains compared to strain AE104 cultivated in the presence of the previously used or new gold solution, respectively. *D* is the distance value as a measure of the statistical significance. *N* ≥3, bold-faced values with *Q* >1.5 and *D* >1.

When the commercially available Au(III) solution was used, the IC_50_ dropped to 5 µM Au(III) in the case of the plasmid-free strain, AE104 (Table 1). A ∆*cop* deletion allele had no longer any effect on resistance, but the ∆*cus* deletion increased gold resistance by 1.56-fold. Therefore, Cus increased Au(III) toxicity, but Cop did not. Deletion of either *cup* or *gig* had no effect. Among the double-deletion strains, ∆*cus ∆gig* and ∆*cop ∆gig* also increased the IC_50_ value twofold compared to the isogenic parent (Table 1). This duplication was also observed with two of the four triple-deletion strains and the quadruple-deletion strains. Components that cooperated to mediate copper resistance interacted here to mediate Au(III) sensitivity. These components affected the periplasmic Cu(I) pool.

As CusCBA and CopA remove Cu(I) from the periplasm by export or oxidation, respectively, and Gig also interacts with periplasmic Cu(I) (10), this indicates that the commercially available Au(III) solutions, in contrast to self-made ones, may inhibit metalation of periplasmic Cu(I)-utilizing proteins, including CopA. Other examples are the Cu-Zn superoxide dismutase, SodC, and the terminal oxidase of the respiratory chain. Components that remove periplasmic Cu(I), or no longer provide it, would consequently increase Au(III) toxicity. Since recovery of gold resistance was not observed, for instance, in the ∆*cop*, ∆*cop ∆cus,* or ∆*cop ∆cus ∆gig* mutants, which all have a ∆*cop* deletion, full metalation of CopA with Cu ions may also have been inhibited. This indicated that upon contact with *C. metallidurans* cells, the commercially available Au(III) solution may release toxic Au species in the periplasm with a higher rate than the previously used self-made solution. Due to the interference with periplasmic Cu(I), this toxic Au species could be an Au(I) species. Re-oxidation of this species to Au(III) may be prevented by an incompletely or wrongly metalated CopA, which enhanced the toxic effect of the commercially available Au(III) solution. Consequently, the impact of this solution on *C. metallidurans* cells was characterized in more detail.

### No appearance of gold nanoparticles in the periplasm

**Fig 2:**
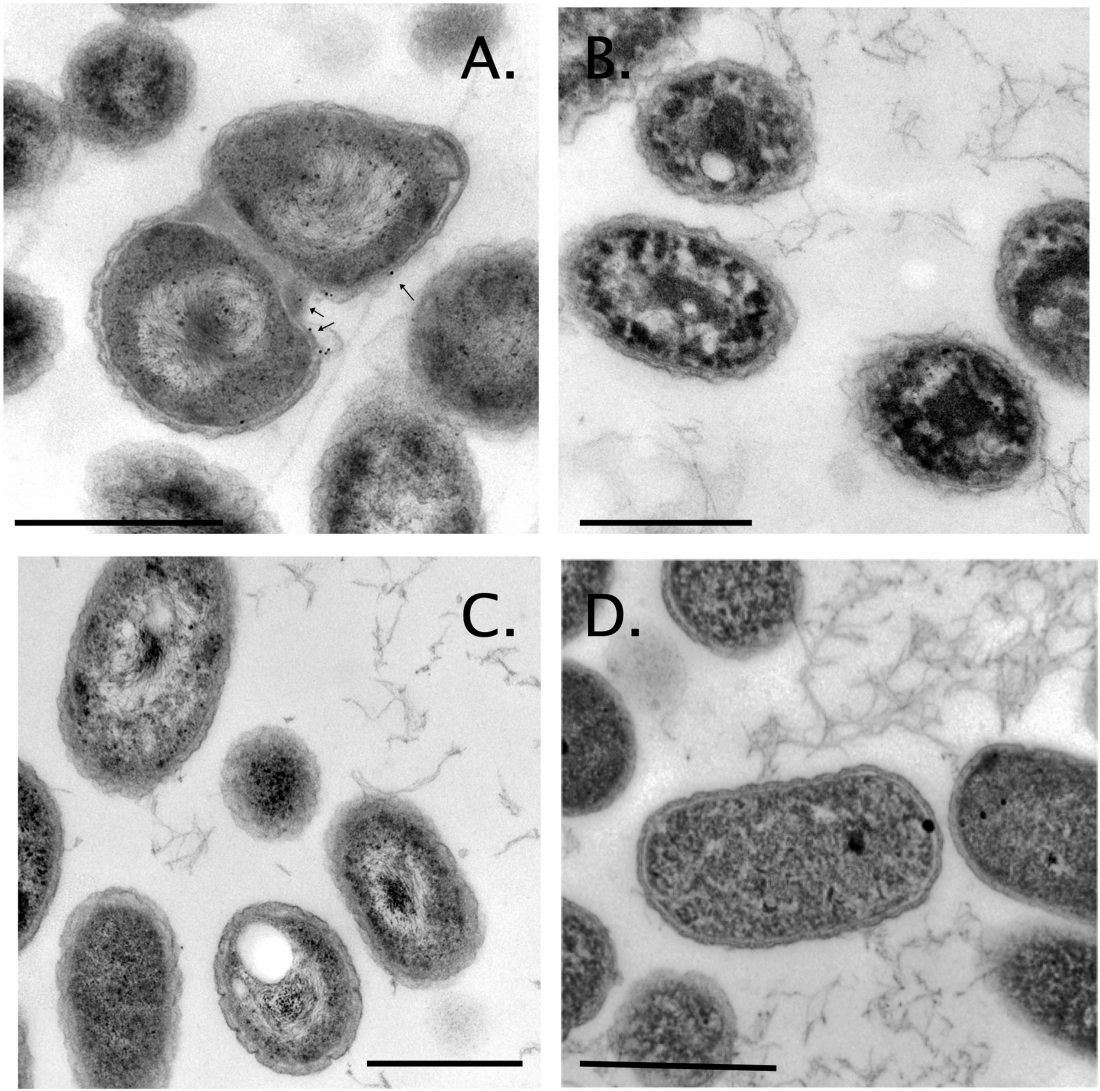
No gold grains visible in cells treated with the new gold chloride solution. Shown are electron microscopic images of *C. metallidurans* AE104 cells (panel A) treated for 72 h with 50 µM previous Au(III) solution, (panel B) 70 h with 50 µM, (panel C) 21 h with 15 µM, and (panel D) 30 min with 50 µM of the new Au(III) solution. Scale bar, 500 nm. Au grains in panel A labeled with an arrow. Photoshop was used to adjust the contrast and brightness.

When *C. metallidurans* was incubated for 3 days in the presence of the self-prepared 50 µM Au(III) solution, Au nanoparticles appeared in the periplasm (Fig. 2A, arrows) as published (5, 6, 8, 9). In contrast, incubation for the same time with the commercially available Au(III) damaged and disintegrated the cells (Fig. 2B). Even incubation for 21 h with 15 µM Au(III) physically damaged the cells (Fig. 2C). When the incubation time in the presence of 50 µM Au(III) was reduced to just 30 min, the cells survived, looked viable but no longer formed Au nanoparticles. This was also observed for lower Au concentrations, and different incubation times tested. Either the cells were damaged or they were not (Fig. 2D). If they were not, then they did not form nanoparticles. The commercially available Au(III) solution was apparently too toxic due to the rapid formation of Au(I) and the subsequent inhibition of the periplasmic Cu(I) homeostasis to allow formation of periplasmic Au nanoparticles. Alternatively, this solution was more toxic than the previously used one because the formation of Au nanoparticles was no longer possible. Moreover, the affected periplasmic Cu(I) homeostasis may lead to an impact on the synthesis of the respiratory chain and, subsequently, to a lower availability of reducing equivalents for the final reduction of Au(I) to Au(0).

### Accumulation of gold atoms by the cells

Confirming a previous report (8), *C. metallidurans* AE104 accumulated 50,000 Au atoms when incubated in the presence of 2.5 µM Au(III) chloride from the self-made solution (Table 2). Due to the synergistic toxicity of Au and Cu, this number increased to 176,000 Au atoms per cell in the additional presence of 100 µM Cu(II) ions. The Cu content of the cells did not change in the presence of 2.5 µM Au(III) when the medium was not amended with Cu ions but increased from 84,000 Cu per cell to 140,000 Cu per cell when 100 µM Cu(II) was added due to the inhibition of the Cu exporter CupA by Au ions (Table 2). Deletion of *cus* increased the Au content of the cells incubated just in the presence of 2.5 µM Au(III) by 88%, deletion of *gig* decreased accumulation of Au atoms in the presence of Cu ions, and deletion of *cup* had no effect.

**TABLE 2:** Metal content of *C. metallidurans* strains in the presence of the previously used gold solution^*a*^

Added metals	1,000 Au/cell		1,000 Cu/cell			
	Au	Au + Cu	0	Au	Cu	Au + Cu
AE104, metals/cell	50.0 ± 7.0	176.0 ± 21.0	18.0 ± 6.0	14.0 ± 6.0	84.0 ± 11.0	140.0 ± 10.0
-Fold AE104 (*Q*, *D*), comparison to reference value						
AE104	1.00 ± 0.14; 0.00	**3.52 ± 0.42; 4.50**	1.00 ± 0.33; 0.00	0.78 ± 0.33; 0.33	**4.67 ± 0.61; 3.88**	**7.78 ± 0.56; 7.63**
AE104, Cu-ind.	**2.10 ± 0.22; 3.06**	**1.86 ± 0.16; 2.87**	n.d.	**2.56 ± 0.22; 2.80**	**3.67 ± 0.67; 2.67**	**4.06 ± 0.44; 3.93**
-Fold AE104 (*Q*, *D*), comparison to AE104 cells under the same conditions						
*∆cusCBAF*	**1.88 ± 0.34; 1.83**	0.91 ± 0.06; 0.47	0.72 ± 0.22; 0.50	0.71 ± 0.14; 0.50	1.46 ± 0.39; 0.89	0.95 ± 0.05; 0.41
*∆cusCBAF*, Cu-ind.	1.20 ± 0.30; 0.49	**2.18 ± 0.62; 1.67**	n.d.	1.15 ± 0.30; 0.39	1.29 ± 0.14; 0.90	1.45 ± 0.07; 2.54
*∆cupCAR*	1.26 ± 0.02; 1.63	0.91 ± 0.34; 0.20	0.61 ± 0.11; 0.88	0.93 ± 0.21; 0.11	1.11 ± 0.20; 0.32	0.97 ± 0.16; 0.13
*∆cupCAR*, Cu-ind.	**0.62 ± 0.04; 2.67**	**1.53 ± 0.06; 3.50**	n.d.	1.48 ± 0.24; 1.47	1.03 ± 0.05; 0.13	1.16 ± 0.19; 0.55
*∆gigPABT*	0.96 ± 0.20; 0.12	**0.65 ± 0.11; 1.53**	0.78 ± 0.28; 0.36	1.29 ± 0.36; 0.36	1.17 ± 0.20; 0.50	0.99 ± 0.11; 0.04
*∆gigPABT*, Cu-ind.	0.72 ± 0.08; 1.53	0.92 ± 0.01; 0.78	n.d.	0.83 ± 0.13; 0.80	**2.09 ± 0.47; 1.67**	1.29 ± 0.01; 2.33

^*a*^ The cells were cultivated in the presence of 2.5 µM Au(III) (previous Au(III) solution), 100 µM Cu(II), both metals, or without additions, and the cellular Au and Cu content was determined by ICP-MS. Moreover, the cells were also pre-incubated with 100 µM Cu(II) in the pre-culture to induce copper resistance operons (“Cu-ind.”). The metal content for strain AE104 is indicated. The Cu content of AE104 cells was compared to that of AE104 cells grown without additions, and the Au content to cells incubated with 2.5 µM Au(III) in the main culture. These reference values are underlined. For the mutant strains, the -fold increase compared to AE104 cells cultivated under the same conditions is indicated. All values are mean -fold increase ± deviation plus the *D*-value after the semicolon. To focus on the major results, significant differences (*Q* >1.5, *Q* <0.67; and *D* >1) are bold faced.; *n* >5; n.d., not done. The values for strain AE104 were obtained under the same conditions as those used for the mutant strains and are published (8).

The cells were also pre-incubated in the presence of Cu ions to induce up-regulation of the copper resistance determinants of the plasmid-free strain AE104. As published (8, 9), this led to a decreased cellular accumulation of Cu and Au atoms in main cultures, which contained both metal ions (Table 2). Absence of *cus* and *cup* increased the cellular Au content under these conditions even more. This indicated that Cus may decrease the cellular Au content in the case of the self-made Au solution in the absence of additional Cu ions, and Gig may increase it in the presence of Cu ions. Because Au(III) strongly inhibits the Cu-exporting P-type ATPase CupA (8), either Au(I) has a different influence on CupA than Au(III) or another product of the *cup* determinant, namely the MerR-type regulator CupR or the metal chaperone CupC, was involved in the decreased accumulation of Au under these conditions.

Since the periplasmic Cu(I) and Au(I) oxidase CopA prevents the accumulation of Au and Cu atoms by the cells (8–10), the actual imported species should be the Cu(I) and Au(I) ions. Should the commercially available Au(III) solution release Au(I) with a higher rate than the previously used solution, the cells should accumulate more Au ions when incubated in the presence of the commercial Au(III) solution. Indeed, when 2.5 µM of this solution was used, the parental strain AE104 contained nearly a million Au atoms per cell, 20 times more compared to the self-made Au(III) solution (Table 3, full data set in Table S1). Because the speciation of Au in the commercially available Au(III) solution caused a higher toxicity of the Au ions, Cu was added at a concentration of only 10 µM when this solution was used. This increased the number of cell-bound Au atoms 1.5-fold, but this was not a significant increase due to the high standard deviation of measurements. Increased toxicity of the commercially available Au(III) solution was associated with an increased accumulation of Au by the cells. The speciation of Au(III) in this solution may indeed have resulted in a higher reduction rate to Au(I) in the periplasm of *C. metallidurans* than that in the previously used one.

**TABLE 3:** Gold and copper content of the mutant cells^*a*^

Strain	2.5 µM Au	2.5 µM Au + 10 µM Cu		
	Au (1,000 /cell)	Au (1,000 /cell)	Cu (1,000 /cell)	*Q* (Au-Cu_Au)
AE104	961 ± 264; 1.0	1,476 ± 296; 1.0	82 ± 27; 1.0	1.54
*ΔgshA* (marker-free)	1,319 ± 148; 1.4	1,870 ± 279; 1.3	75 ± 7; 0.9	1.42
*∆gshA* (disrupted)	1,028 ± 228; 1.1	***913 ± 98; 0.6***	***40 ± 5; 0.5***	0.89
*∆gshA* (pBBR::*gshA*)	1,196 ± 245; 1.2	1,316 ± 138; 0.9	92 ± 15; 1.1	1.10
*Δcop*	1,003 ± 211; 1.0	1,678 ± 268; 1.1	**280 ± 112; 3.4**	**1.67**
*Δcop Δcup ∆gshA*	828 ± 385; 0.9	***894 ± 105; 0.6***	**209 ± 100; 2.5**	1.08
*Δcop Δcup ∆gshA*(pBBR)	983 ± 89; 1.0	1,363 ± 227; 0.9	**248 ± 56; 3.0**	1.39
*Δcop Δcup ∆gshA*(pBBR::*gshA*)	1,321 ± 143; 1.4	1,191 ± 226; 0.8	**314 ± 57; 3.8**	0.90
*Δcop Δcup Δcus*	893 ± 258; 0.9	1,435 ± 174; 1.0	**326 ± 56; 4.0**	1.61
*Δcop Δcup Δcus ∆gshA*	910 ± 146; 0.9	1,147 ± 96; 0.8	**366 ± 42; 4.5**	1.26
*Δcop Δcup Δcus ∆gshA*(pBBR)	797 ± 58; 0.8	***908 ± 47; 0.6***	**262 ± 18; 3.2**	1.14
*Δcop Δcup Δcus ∆gshA*(pBBR::*gshA*)	1,349 ± 214; 1.4	1,481 ± 308; 1.0	**351 ± 43; 4.3**	1.10
*Δcop Δcup Δgig*	925 ± 255; 1.0	1,454 ± 237; 1.0	**272 ± 50; 3.3**	1.57
*Δcop Δcup Δgig ∆gshA*	962 ± 349; 1.0	***795 ± 192; 0.5***	**139 ± 29; 1.7**	0.83
*Δcop Δcup Δgig ∆gshA*(pBBR)	965 ± 47; 1.0	1,220 ± 112; 0.8	**231 ± 28; 2.8**	1.26
*Δcop Δcup Δgig ∆gshA*(pBBR::*gshA*)	**2,008 ± 210; 2.1**	**1,636 ± 153; 1.1**	**321 ± 76; 3.9**	0.81
*Δcop Δcus*	1,077 ± 239; 1.1	1,822 ± 304; 1.2	**403 ± 179; 4.9**	1.69
*Δcop Δcus ∆gshA*	1,238 ± 442; 1.3	1,047 ± 87; 0.7	**440 ± 43; 5.4**	0.85
*Δcop Δcus ∆gshA*(pBBR)	927 ± 58; 1.0	1,498 ± 291; 1.0	**331 ± 218; 4.0**	1.62
*Δcop Δcus ∆gshA*(pBBR::*gshA*)	1,348 ± 80; 1.4	1,626 ± 299; 1.1	**574 ± 181; 7.0**	1.21
*Δcop Δcus Δgig*	1,079 ± 213; 1.1	1,653 ± 169; 1.1	**280 ± 52; 3.4**	1.53
*Δcop Δgig*	928 ± 216; 1.0	1,598 ± 318; 1.1	**250 ± 71; 3.0**	1.72
*Δcup*	944 ± 145; 1.0	1,470 ± 334; 1.0	99 ± 37; 1.2	1.56
*Δcup ∆gshA*	993 ± 265; 1.0	987 ± 241; 0.7	***52 ± 2; 0.6***	0.99
*Δcup ∆gshA*(pBBR)	1,111 ± 142; 1.2	1,501 ± 193; 1.0	79 ± 22; 1.0	1.35
*Δcup ∆gshA*(pBBR::*gshA*)	1,031 ± 104; 1.1	1,064 ± 126; 0.7	102 ± 25; 1.2	1.03
*Δcus*	741 ± 208; 0.8	1,188 ± 199; 0.8	90 ± 31; 1.1	1.60

^*a*^ The metal content was determined by ICP-MS. The full data set is provided in Table S1. The metal content of the cells was determined by ICP-MS in cells cultivated in the presence of 2.5 µM Au(III)HCl_3_ and 2.5 µM Au(III)HCl_3_ plus 10 µM Cu(II). The Cu content is provided only for the Au/Cu-cultivated cells. The mean values of ≥3 determinations is provided with the deviation and is followed by the ratio of this value with the AE104 result. Bold-faced letters indicate significant differences (*D* >1, *Q* >1.5, or *Q* <0.67), in italics if down-regulated. The last row compares the Au contents of the cells cultivated in the presence of Au(III) with and without added Cu ions. Light gray field: Cu content down-regulated compared to the ∆*cop* strain, and medium gray field up-regulated. *C. metallidurans* strain AE104 cultivated in non-amended medium contained 8,070 ± 4,000 Cu per cell. This number did not change in the mutants and also not in the presence of 2.5 µM new Au solution. The mean value of the cellular Cu content of all mutants in non-amended and Au-containing medium was 7,230 ± 2,200 Cu per cell.

To study the interaction of Au complexes with copper resistance determinants in more detail, single- and multiple-deletion mutants were constructed up to the quadruple-deletion strain ∆*cup ∆cop ∆cus ∆gig*. None of these mutant strains accumulated more Au atoms from 2.5 µM of the commercially available solution than the parent strain (Table 3; Table S1), neither in the absence nor presence of added Cu ions. The ∆*cop* single mutant and all ∆*cop* mutants with additional deletions, as well as the ∆*cus* and *∆cup* single mutants, displayed an increased Au content in Au(III)/Cu(II) medium compared to Au(III) only. This indicated that even in the presence of the commercially available Au(III) solution, *C. metallidurans* accumulated more Au atoms in the presence of Cu ions compared to in their absence. The absence of the Cop system, centered around the Cu-dependent Cu(I)/Au(I) oxidase CopA, caused a decrease in the cellular Au content but only in the presence of added Cu, which compensated the inhibition of the periplasmic Cu(I) homeostasis by Au ions. By interfering with the periplasmic Cu(I) homeostasis, the high amount of Au(I) generated from the commercial Au(III) species in the periplasm indeed inhibited the Cu-dependent oxidation of Au(I) in this compartment by CopA. Subsequently, only the interaction of *C. metallidurans* with the commercially available Au(III) solution was characterized.

### Synergistic toxicity of gold and copper ions

Due to the inhibition of the CupA Cu(I)-efflux pump of the inner membrane, the presence of Au complexes synergistically decreases copper resistance of *C. metallidurans* (8). To learn more about the Cu- and Au resistance mechanisms, this inhibition of copper resistance by Au complexes was also studied with the commercially available Au(III) solution using the parental strain AE104 and its isogenic deletion mutants (Table 4). The presence of 2.5 µM Au(III) did not increase the cellular Cu content in non-amended medium as determined by ICP-MS measurements. This number was 8,070 ± 4,000 Cu per cell in strain AE104 in the absence of Au complexes and was also similar in all mutants in media with and without Au but without added Cu; the mean value was determined to be 7,230 ± 2,200 Cu per cell. In the absence of Au, the cellular Cu content increased from 20,000 Cu per cell (1 µM added) to 33,000 Cu per cell [25 µM added; (10)] to 82,000 Cu per cell in the presence of 10 µM Cu(II) plus 2.5 µM Au(III) (Table 3). This number was more than threefold higher in all ∆*cop* mutants, emphasizing the importance of the periplasmic Cu(I) oxidase, CopA, in decreasing the cellular accumulation of Cu in the presence (Table 3; Table S1) and absence (10) of Au complexes.

**TABLE 4:** Influence of gold complexes on copper resistance^*a*^

Strain	+*gshA*			*gshA* disrupted			Comparison ± GSH	
	IC_50_ (−Au)	IC_50_ (+Au)	*Q* (±Au)	IC_50_ (−Au)	IC_50_ (+Au)	*Q* (±Au)	*Q* (GSH − Au)	*Q* (GSH + Au)
	µM Cu(II)	µM Cu(II)	-Fold	µM Cu(II)	µM Cu(II)	-Fold	-Fold	-Fold
AE104	648 ± 48	406 ± 50	**1.60**	477 ± 37	269 ± 34	**1.78**	**0.74**	**0.66**
*Δcop*	370 ± 52	337 ± 21	1.10	235 ± 59	101 ± 14	**2.33**	**0.63**	**0.30**
*Δcup*	16.4 ± 2.7	16.2 ± 3.7	1.01	5.6 ± 1.7	4.6 ± 0.5	1.21	**0.34**	**0.28**
*Δcus*	819 ± 36	705 ± 30	**1.16**	729 ± 84	480 ± 24	**1.52**	0.89	**0.68**
*Δgig*	635 ± 26	509 ± 72	**1.25**	457 ± 44	172 ± 39	**2.66**	**0.72**	**0.34**
*Δcop Δcup*	1.9 ± 0.8	1.8 ± 0.5	1.05	0.5 ± 0.1	0.4 ± 0.0	1.32	**0.27**	**0.22**
*Δcop Δcus*	203 ± 23	174 ± 16	1.17	25.8 ± 1.1	18.7 ± 2.1	**1.38**	**0.13**	**0.11**
*Δcop Δgig*	498 ± 35	367 ± 43	**1.36**	124 ± 16	82.9 ± 5.6	**1.49**	**0.25**	**0.23**
*Δcus Δgig*	638 ± 29	527 ± 46	**1.21**	446 ± 48	268 ± 60	**1.66**	**0.70**	**0.51**
*Δcup Δgig*	9.2 ± 3.0	14.1 ± 3.7	0.65	4.4 ± 0.9	8.9 ± 2.0	**0.50**	**0.48**	0.63
*Δcup Δcus*	4.4 ± 0.7	5.7 ± 1.5	0.77	5.5 ± 1.6	8.7 ± 3.4	0.63	1.25	1.52
*Δcup Δcus Δgig*	3.6 ± 0.7	5.1 ± 0.9	0.69	0.7 ± 0.2	1.8 ± 0.4	**0.39**	**0.20**	**0.36**
*Δcop Δcus Δgig*	190 ± 15	145 ± 5	**1.31**	23.1 ± 1.8	16.5 ± 0.7	**1.40**	**0.12**	**0.11**
*Δcop Δcup Δgig*	1.6 ± 0.5	2.9 ± 0.5	**0.53**	0.6 ± 0.1	0.5 ± 0.1	1.12	**0.37**	**0.18**
*Δcop Δcup Δcus*	1.7 ± 0.5	2.2 ± 0.3	0.79	0.5 ± 0.1	0.6 ± 0.0	0.80	**0.26**	**0.26**
*Δcop Δcup Δcus Δgig*	1.7 ± 0.4	1.8 ± 0.2	0.89	0.6 ± 0.0	0.5 ± 0.1	1.15	**0.34**	**0.26**

^*a*^ The IC_50_ value was calculated from dose-response curves, *n* ≥3. Gold (commercially available Au(III) tetrachloride) was added at a concentration of 2.5 µM if indicated, all values with *D* >1 in bold, strains with increased copper resistance in the presence of Au on a gray field.

In the presence of Au complexes, copper resistance of the parental strain, AE104, decreased 1.6-fold (Table 4). The influence of the resistance factors on copper resistance decreased in the order Cup > Cop > Cus > Gig (Table 4) (10). The synergistic toxicity in the presence of Au decreased in the opposite order. There was no longer an influence of Au on the already low level of copper resistance of the ∆*cup* mutant. Subsequent deletion of other resistance determinants in the ∆*cup* mutant decreased copper resistance even further, and there was no longer a synergistic toxicity of Au ions on copper resistance present. In contrast, Au ions increased copper resistance to a small extend in all ∆*cup* mutants, except the single ∆*cup* mutant and the ∆*cop ∆cup* double mutant (Table 4, gray fields). The differences between the presence and absence of Au were significant (*D* >1) in the case of a ∆*cop ∆cup ∆gig* mutant (Table 4), which only contained the Cus system as residual copper resistance system. Cus, or expression of *cus*, seems to be activated by Au complexes to increase copper resistance, although on a very low level.

The ∆*cop* mutant barely had an effect on the synergistic toxicity in the presence of Au. This was evidence that Au complexes indeed may inhibit the Cu-dependent activity of the Cu(I) and Au(I) oxidase CopA. With additional deletions of *cus* or *gig*, synergistic toxicity increased again to the ∆*cop ∆gig* mutant. Resistance in the presence of Au decreased 1.36-fold compared to the absence of Au, which was a similar value as the 1.21-fold decrease in the ∆*cus ∆gig* mutant (Table 4). The 1.31-fold decrease in copper resistance of the ∆*cop ∆cus ∆gig* triple mutant in the presence of Au was on a similar level. This mutant retained the Cup system as the only remaining copper resistance determinant. Because the Cu(I)-exporting P_IB1_-type ATPase, CupA, was inhibited by Au(III) complexes *in vitro* (8), this explained the synergistic Au and copper toxicity in this triple and both double mutants, although a complete inhibition of CupA in the triple mutant would have been expected to decrease the copper resistance level down to that of the quadruple mutant (Table 4). On the one hand, this indicated that CupA was indeed inhibited also *in vivo* by the Au species arriving in the cytoplasm, most likely an Au(I) species. On the other hand, the other *cup* products, CupR or CupC, may have a function in copper resistance that was not connected to their proposed function of *cup* regulator or delivery of Cu(I) to CupA for efflux, respectively.

### Regulation of *cus* by gold complexes

As published, a *cus-lacZ* reporter operon fusion was up-regulated in strain AE104 by increasing copper concentrations fourfold to 20 U/mg dry mass [Fig. S2, (10)]. Deletion of the *cop* determinant, encoding the periplasmic Cu(I) and Au(I) oxidase CopA, increased expression of *cus-lacZ* fourfold. This indicated that *cus* expression was regulated by sensing periplasmic Cu(I) ions. Gig and glutathione had no influence on copper-mediated *cus* induction.

**Fig 3:**
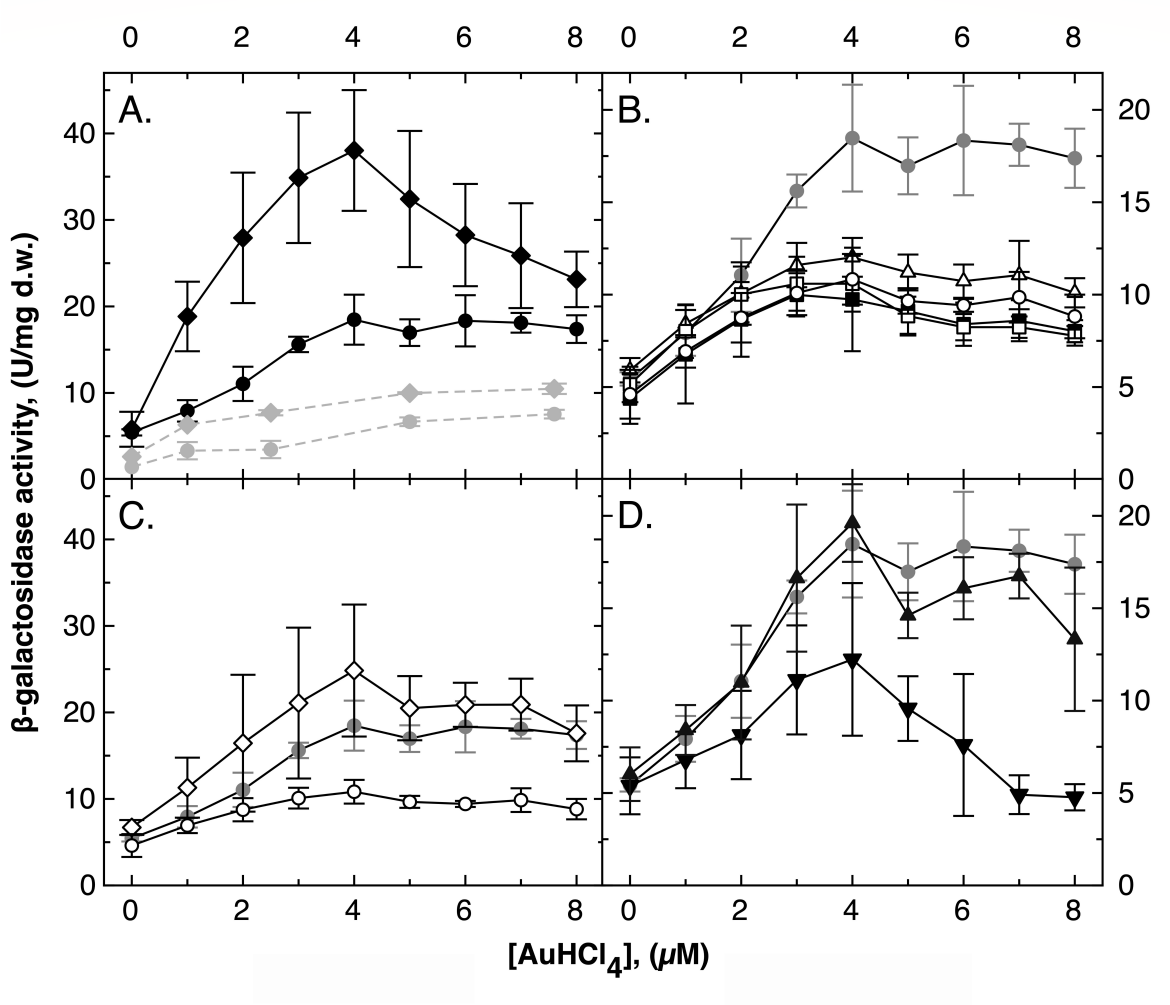
Regulation of *cus* by gold complexes. Reporter gene fusions with the *lacZ* gene were constructed with the *cus* operon in various mutant backgrounds. The strains were incubated in the presence of increasing Au concentrations, and the beta-galactosidase activity was determined. In all panels: strain AE104 (closed circles,●), black in panel A, mid-gray in all other panels for comparison. Panel A: ∆*cop* (closed diamonds,◆). Moreover, results of previous experiments performed with the self-made Au(III) solution were shown in light gray symbols and dashed lines, AE104 (closed circles,●) and ∆*cop* (closed diamonds,◆). Panel B: ∆*cup* (closed squared,■), ∆*cup ∆gig* (open squares,□), ∆*cop ∆cup* (open triangles,△), and ∆*cop ∆cup ∆gig* (open circles,○). Panel C compares ∆*cop ∆gig* (open diamonds,◇) with the parent and *∆cop* ∆*cup* ∆*gig* using the same scale of the *y*-axis as in panel A. Panel D: ∆*gshA* (closed inverted triangles,▼) and ∆*gig* (closed triangles,▲). Deviations shown (*n* ≥3). The published induction experiment with Cu ions (10) is shown for comparison as Fig. S2.

Increasing the concentration of Au complexes (Fig. 3) up-regulated the *cus-lacZ* expression to a level of about 20 U/mg dry mass, albeit with only 4 µM Au(III)HCl_4_ compared with 100 µM Cu(II) (Fig. 3A; Fig. S2). Again, *cus-lacZ* expression was more strongly up-regulated in a ∆*cop* mutant (two times with Au compared to four times with Cu). Since CopA oxidizes periplasmic Au(I), the *cus* inducer should be an Au(I) species.

Compared to a previous measurement that used the self-made Au(III) solution (Fig. 3A, light-gray symbols), up-regulation of *cus-lacZ* was much stronger when the commercially available Au solution was used. This solution released more of the inducing Au(I) species in the periplasm than the self-made solution.

In contrast to copper-dependent *cus* regulation, presence of *gig* was required for enhanced up-regulation of *cus-lacZ* but only in the ∆*cop* mutant and no other mutant background (Fig. 3). The periplasmic oxidase CopA removed the inducing Au(I) species, but Gig seem to be involved in the production of the fully active periplasmic enzyme, as has been considered before (10).

All mutants with a ∆*cup* deletion still doubled *cus-lacZ* expression from 0 to 4 µM Au(III), but expression did not increase further at higher Au concentrations (Fig. 3B). A similar influence of ∆*cup* on Cu-mediated *cus-lacZ* expression had been discussed as the result of a toxic effect because ∆*cup* mutants are very copper sensitive due to the missing Cu(I) exporter CupA [Fig. S2, (10)]. Since the beta-galactosidase remains at the same level with increasing Au concentration, this could also indicate a regulatory influence of the products of the *cup* determinant in *cus* expression as an alternative explanation. Together with the effects of at least one *cup* product on the cellular accumulation of Au in Cu-induced cells and the contribution to resistance to the synergistic Au and copper toxicity, this indicated that CupR may have additional regulatory functions besides controlling expression of *cup*. Up-regulation of *cus-lacZ* by Au also required glutathione at 7 and 8 µM Au(III) (Fig. 3B). Such an effect was not observed with Cu ions (Fig. S2, (10)].

The enhanced up-regulation of *cus-lacZ* with increasing Au(III) concentrations in the ∆*cop* mutant compared to the parent indicated that *cus* was regulated by periplasmic Au(I) species at concentrations up to 4 µM Au(III) and that its regulation may be influenced by products of CupR and by glutathione at higher Au concentrations. Similar to Cu(I) and Ag(I), Au(I) may bind to the periplasmic domain of a sensory histidine kinase such as CusS from *E. coli* (15–18). But there were no genes for a two-component regulatory system in the vicinity of *cus* (10), and the only two-component regulatory system related to the *cusRS* system from *E. coli* in the genome of the plasmid-free strain AE104 was the *copR_2_S_2_* genes (18, 19).

To analyze whether this system controls *cus* expression, the *copS_2_* gene for a sensory histidine kinase, the *copR_2_* gene for its response regulator, and both genes were deleted in *C. metallidurans* AE104. As published (10), copper resistance was increased in the ∆*cus* mutant in a *cop*-dependent manner because lacking removal of periplasmic Cu(I) in this mutant should induce up-regulation of *cop* expression (Fig. S3A). Copper resistance was strongly decreased in the ∆*copS_2_* mutant but increased again to a higher resistance level than that of the parent in the ∆*copR_2_S_2_* mutant, while resistance of the ∆*copR_2_* mutant was similar to that of the parent. The copper resistance level of the ∆*copS_2_* mutant was similar to that of the ∆*cop ∆cus* double mutant and not of the ∆*cop* single mutant. CopS_2_ was not only involved in *cop* but also in *cus* expression. Additional deletion of *copR_2_* increased resistance again to a level between the parent strain and its *∆cus* mutant. The strong decrease of copper resistance in the ∆*copS_2_* mutant was *copR_2_* dependent, and deletion of both genes seems to result in an up-regulation of *cop* or *cus*.

Up-regulation of *cus* with increasing Cu concentrations was strongly increased in the ∆*copABCD* deletion mutant and to a similar level in the ∆*copS_2_* mutant (Fig. S3A), especially at Cu concentrations between 50 and 100 µM Cu(II). CopS_2_ was involved in *cop* regulation. In the single ∆*copR_2_* and the ∆*copR_2_S_2_* double mutant, the *cusF-lacZ* expression level was also up-regulated compared to the parent, but the expression level increased up to 300 µM Cu(II). But Cu-dependent up-regulation of *cus* expression was still possible. CopR_2_S_2_ were involved in the regulation of *cus* expression but not essential for this process.

Unexpectedly, a *copABCD-lacZ* fusion was not up-regulated with increasing Cu concentrations and deletion of *copS_2_, copR_2_,* or both genes (Fig. S3). Together with a possible involvement of CupR, this indicated that CopR_2_S_2_ were part of a complicated regulatory network that controls Cu- and Au-mediated regulation of *cup, cus,* and *cop*. Nevertheless, the sensory histidine kinase CopS_2_ was not useful as part of a reporter system able to sense the periplasmic Au(I) concentration.

### Regulation of *gig* by gold complexes

As also demonstrated previously using a *gig-lacZ* operon fusion (10), expression of this reporter increased to about 60 U/mg dry mass in the parental strain by increasing Cu concentrations and to nearly 250 U/mg dry mass in the ∆*cop* mutant. Expression levels of nearly 400 U/mg dry mass were achieved in a ∆*cop ∆cus* strain (Fig. S4). Deletion of *cup* decreased activation of *gig* at higher Cu (Fig. S4C) as well as GSH at lower (Fig. S4B) concentrations. A *cup* product, most likely CupR, seemed to be a master regulator of *gig* and *cus* at higher Cu concentrations, GSH or a product of GSH may sequester the *gig* inducer, which is removed by CopA and Cus and therefore periplasmic Cu(I).

**Fig 4:**
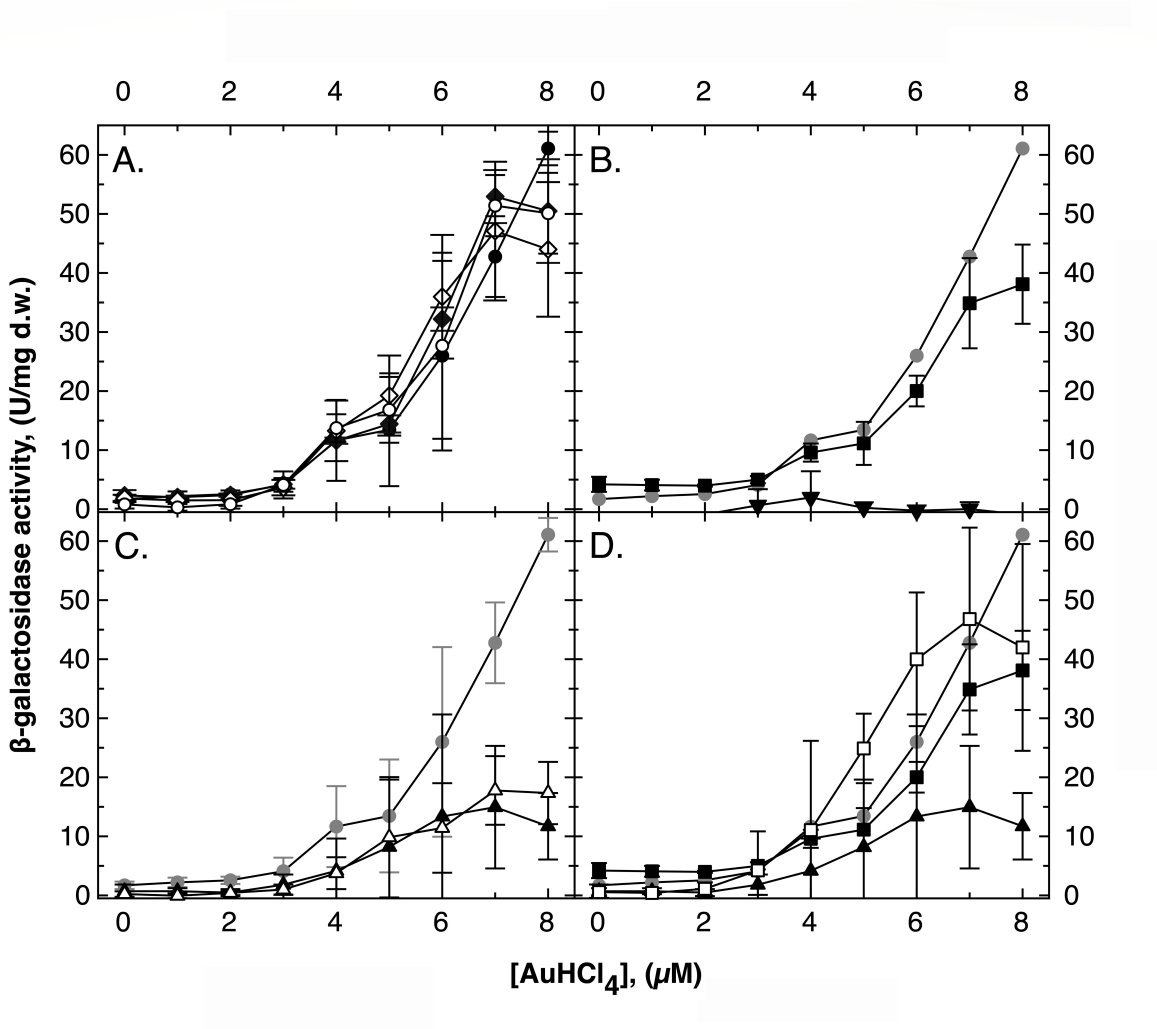
Regulation of *gig* by gold complexes. Reporter gene fusions with the *lacZ* gene were constructed with the *gig* operon in various mutant backgrounds. The strains were incubated in the presence of increasing Au concentrations, and the beta-galactosidase activity was determined. In all panels: strain AE104 (closed circles,●), black in panel A, gray in the other panels for comparison. Panel A: ∆*cop* (closed diamonds,◆), ∆*cop ∆cus* (open diamonds,◇), ∆*cop ∆cus ∆cup* (open circles,○). Panel B: ∆*gshA* (closed inverted triangles,▼) and ∆*cup* (closed squared,■). Please note the essential contribution of GSH to Au-dependent up-regulation of *gig* compared to the Cu-dependent up-regulation (Fig. S3). Panel C: comparison of ∆*cop ∆cup* (open triangles,△) with ∆*cus* (closed triangles,▲). Panel D: ∆*cup* (closed squared,■), ∆*cus* (closed triangles,▲), and ∆*cup ∆cus* (open squares,□). The published induction experiment with Cu ions (10) is shown for comparison as Fig. S4.

In contrast, there was no difference in *gig-lacZ* up-regulation by Au complexes in the ∆*cop* and ∆*cop ∆cus* mutants. Only three deletions influenced Au-dependent up-regulation of *gig*: ∆*cus, ∆cop ∆cup,* and ∆*gshA* (Fig. 4). The deletions of ∆*cus* and *∆cop ∆cup* decreased up-regulation of *gig-lacZ* by Au complexes, but any additional deletion in the ∆*cus* or in the *∆cop ∆cup* background led back to the parent level. A Cus product was required to generate the *gig* inducer but only in the presence of the Cop and the Cup system. On the other hand, Cop and Cup were needed but only in the presence of Cus. This indicated that a complicated interaction of the three copper resistance determinants, encoding efflux pumps, periplasmic and cytoplasmic binding proteins, a possible copper uptake system, and the regulator CupR, was required to generate the *gig* inducer from Au(III) complexes. But most important was glutathione, which was essential for this process. The *gig* inducer was derived from the Au(III) complexes by interaction with glutathione or its periplasmic products.

### Gold and copper decrease the cellular glutathione content

Glutathione was essential to produce the *gig* inducer from Au(III) complexes (Fig. 4). To understand the influence of GSH on Au resistance and resistance to synergistic Au/Cu toxicity, the glutathione content of *C. metallidurans* parent strain AE104 and its mutants was determined in the presence of Au complexes and additionally Cu ions (Table 5). As published, the ∆*cus* mutant contained a lower GSH content even in the absence of added Cu ions (10).

**TABLE 5:** Glutathione content of *C. metallidurans* mutant cells^*a*^

Bacterial strains	Glutathione content (nmol GSH/mg protein)		
	No addition	+ Au	+ Au + Cu
AE104	962 ± 109; 100%	**795 ± 39; 83%**	709 ± 55; 89%
*Δcop*	978 ± 122; 102%	894 ± 91; 91%	834 ± 56; 93%
*Δcup*	894 ± 50; 93%	772 ± 99; 86%	679 ± 107; 88%
*Δcus*	**373 ± 46; 39%**	**262 ± 45; 70%**	286 ± 52; 109%
*Δgig*	883 ± 133; 92%	863 ± 77; 98%	796 ± 67; 92%
*Δcop Δcup*	1,041 ± 169; 108%	905 ± 113; 87%	**697 ± 55; 77%**
*Δcop Δcus*	1,034 ± 118; 108%	801 ± 39; 77%	**597 ± 104; 75%**
*Δcop Δgig*	956 ± 139; 99%	889 ± 61; 93%	839 ± 107; 94%
*Δgig Δcus*	957 ± 66; 99%	**799 ± 82; 83%**	665 ± 103; 83%
*Δcup Δgig*	857 ± 166; 89%	796 ± 51; 93%	721 ± 8; 91%
*Δcup Δcus*	805 ± 163; 84%	742 ± 69; 92%	610 ± 114; 82%
*Δcup Δcus Δgig*	738 ± 175; 77%	739 ± 60; 100%	**578 ± 14; 78%**
*Δcop Δcus Δgig*	746 ± 160; 78%	785 ± 81; 105%	**357 ± 87; 46%**
*Δcop Δcup Δgig*	735 ± 186; 76%	780 ± 122; 106%	**361 ± 142; 46%**
*Δcop Δcup Δcus*	823 ± 237; 86%	777 ± 164; 94%	**312 ± 89; 40%**
*Δcop Δcup Δcus Δgig*	**721 ± 124; 75%**	729 ± 225; 101%	**280 ± 63; 38%**
Strains containing pBBR::*gshA*			
AE104*; +gshA*	822 ± 157; 85%	669 ± 105; 81%	683 ± 60; 102%
***ΔgshA**; +gshA*	1,077 ± 165; 131%^*b*^	1,019 ± 165; 95%	1,135 ± 178; 111%
*∆gshA; +gshA*	**258 ± 88; 31%** ^*b*^	360 ± 55; 140%	366 ± 89; 102%
*Δcop ∆gshA; +gshA*	**475 ± 141; 39%**	501 ± 66; 105%	518 ± 82; 103%
*Δcup ∆gshA; +gshA*	**377 ± 57; 42%**	**498 ± 46; 132%**	484 ± 65; 97%
*Δgig ∆gshA; +gshA*	**499 ± 71; 56%**	524 ± 101; 105%	506 ± 90; 97%
*Δcop Δcup ∆gshA; +gshA*	**518 ± 66; 50%**	720 ± 138; 139%	**450 ± 50; 63%**
*Δcop Δcus ∆gshA; +gshA*	**349 ± 103; 34%**	407 ± 123; 116%	**231 ± 42; 57%**
*Δcop Δgig ∆gshA; +gshA*	**568 ± 134; 59%**	695 ± 150; 122%	738 ± 67; 106%
*Δgig Δcus ∆gshA; +gshA*	**382 ± 84; 40%**	511 ± 112; 134%	504 ± 98; 99%
*Δcup Δgig ∆gshA; +gshA*	**384 ± 28; 45%**	477 ± 76; 124%	563 ± 94; 118%
*Δcup Δcus ∆gshA; +gshA*	**386 ± 68; 48%**	489 ± 83; 127%	521 ± 89; 107%
*Δcup Δcus Δgig ∆gshA; +gshA*	**349 ± 44; 47%**	340 ± 56; 97%	325 ± 28; 96%
*Δcop Δcus Δgig ∆gshA; +gshA*	**482 ± 45; 65%**	530 ± 63; 110%	**296 ± 43; 56%**
*Δcop Δcup Δgig ∆gshA; +gshA*	499 ± 65; 68%	540 ± 98; 108%	416 ± 69; 77%
*Δcop Δcup Δcus ∆gshA; +gshA*	**375 ± 35; 45%**	344 ± 23; 92%	**221 ± 32; 64%**
*Δcop Δcup Δcus Δgig ∆gshA; +gshA*	617 ± 26; 86%	696 ± 116; 113%	**155 ± 39; 22%**

^*a*^ The glutathione content of the cells was determined without addition of Au, with the addition of 2.5 µM Au(III)HCl_4_, and additionally with 10 µM Cu(II). *n* ≥3, deviations indicated. Comparisons (in %) are (i) first row without additions and not complemented in *trans* with *gshA* mutants compared to parent AE104; (ii) first row complemented strains in the lower half of the table with the respective not complemented strain above; (iii) strains with Au with those without Au; and (iv) strains with Au and Cu with those with Au. Bold-faced numbers indicate differences with *D* >1. Bold-faced ∆*gshA* indicates a marker-free deletion, all other ∆*gshA* strains contained an interrupted gene.

^*b*^ Comparison with AE104-carrying pBBR1MCS-3::*gshA*.

Adding just Cu ions decreased the GSH content in ∆*cus ∆cop ∆gig* mutants, increased it in the ∆*cup ∆gig* mutants but not the parent strain, and did not diminish the low GSH content of the ∆*cus* mutant even further (10). Addition of Au complexes decreased the GSH content in the parent, the ∆*cus*, and the ∆*cus ∆gig* mutants (Table 5). In other single- and double-deletion mutants, the GSH content was also lowered, but the differences with and without Au were not statistically significant. The GSH content of the triple and the quadruple mutants was unchanged compared with other single and double mutants, but these already had a slightly lower GSH content in the absence of Au.

Gold plus Cu ions resulted in a decreased GSH content of the quadruple, all triple, and half of the double mutants, namely ∆*cop ∆cup, ∆cop ∆cus,* and ∆*cup ∆gig* (Table 5). In the remaining double mutants, the GSH content decreased but not to a significantly different value. The GSH content was lowest in the quadruple mutant and was at a similar level in the triple mutant containing only the Gig system. It increased compared to these two via the *cus*- and *cup*-only to the *cop*-only triple mutant. The lowest GSH content of all these strains, however, was found in the ∆*cus* mutant in the presence of Au complexes, and this content did not decrease further in the presence of Au and Cu.

The cellular GSH content was decreased in the ∆*cus* mutant, in multiple deletion mutants in the absence of challenging Cu and Au ions, in the presence of Au complexes in the parental strain, single, and double mutants, and even more in the presence of both metals. Under these conditions, less GSH was synthesized, and more was degraded or exported from the cell.

The *gshA* gene was deleted in strain AE104 (parental) and its isogenic mutants. None of these ∆*gshA* strains contained measurable levels of GSH (10). The ∆*gshA* derivatives were complemented in *trans* with a plasmid pBBR1MCS-3 derivative with an integrated *gshA* gene under the control of its own promoter. Additionally, a marker-free *∆gshA* mutant of the parent was also complemented. Such a mutant could only be obtained from the parental strain but not for any of the mutants.

Complementation of the marker-free ∆*gshA* deletion mutant of parent AE104 resulted in a complete restoration of the GSH content, but complementation of all strains with a chromosomally interrupted *gshA* gene resulted in lower levels of GSH of between 31% and 86% of the level of the respective parental strain with intact *gshA* gene (Table 5). This could have been the result of different growth conditions of the strains in the pre-cultures, the presence of tetracycline to select for the vector and additionally of kanamycin for the gene disruption. Addition of Au complexes did not change the GSH levels in the ∆*gshA* mutants (Table 5). Addition of Cu and Au decreased the GSH content of five strains significantly, the complemented ∆*gshA* mutants of ∆*cop ∆cup, ∆cop ∆cus, ∆cop ∆cus ∆gig*, ∆*cop ∆cup ∆cus,* and of the quadruple mutant. Including a statistically non-significant 77% decreased level in the derivative of the ∆*cop ∆cup ∆gig* triple mutant, these were all derivatives of the ∆*cop* mutant with one or more additional deletions, the exception being the ∆*cop ∆gig* strain. The GSH levels in the quintuple mutant in the presence of Au and Cu were reduced to 16% of the level of the unchallenged parental strain, AE104 (Table 5).

Complementation in *trans* of strains with an interrupted *gshA* gene resulted in a restored synthesis of GSH but not to the same level as in the isogenic strains with the wild-type *gshA* gene or the complemented parent strain with a marker-free deletion in *gshA*. Au did not decrease this level further, but Au plus Cu did in most of the ∆*cop* deletion strains.

### Metal content of the ∆*gshA* mutant strains

The Au content of various strains with mutations in copper resistance systems was unchanged compared to the parental strain when incubated in the presence of Au (Table 3; Table S1). Deletion of ∆*gshA* or in *trans* complementation with *gshA* of these mutants did not change this; however, there were two exceptions. A complemented ∆*cop ∆cup ∆gig ∆gshA* mutant displayed a twofold higher Au level (Table 3). This strain possessed only a slightly higher GSH level compared to all other triple mutants in the presence of Au, and the Cus system was the sole system operating for copper resistance in these strains. Secondly, a vector control of a ∆*gig ∆cus ∆gshA* mutant contained a 1.6-fold elevated Au level. The Au level of this mutant without the vector pBBR was 1.3-fold higher than the parent, which was not a statistically significant result.

In the presence of Au and Cu, the Au content of five mutants was decreased about by half. These mutants included the ∆*gshA* strain, ∆*cop ∆cup ∆gshA*, ∆*cus ∆gshA*, and the ∆*cop ∆cup ∆cus ∆gshA*(pBBR) strain. Considering that the respective strains with or without the vector plasmid pBBR did not exhibit any changes in their Au content, these differences were not taken into further consideration. The same was true for the decreased Cu content in the presence of Au in the ∆*gshA, ∆cup ∆gshA,* or the ∆*gig ∆cus ∆gshA* mutants (Table 3; Table S1).

The content of Cu in the presence of Au was increased between 1.7- and 7-fold in all ∆*cop* mutants compared to the parent strain (Table 3; Table S1), which was reminiscent of the level after growth in the presence of just Cu (10). Compared to the *∆cop* mutant, the Cu content in the presence of Au ions was lower in the ∆*cop ∆cup ∆gig ∆gshA* mutant, which displayed increased Au contents for the same strain when they were *trans* complemented. In a strain that contains only the Cus system, absence of GSH resulted in a decreased Au and Cu content under conditions of synergistic toxicity, but the levels were increased again when the strain was complemented in *trans* with *gshA*. Also, compared to the ∆*cop* mutant, the Cu content in the presence of Cu and Au was significantly increased, with the highest measured content being more than 500,000 Cu, in addition to the 1.6 million Au atoms per cell in the complemented *∆cop ∆cus ∆gshA* mutant. The GSH content of this mutant was the third-lowest level measured. In this strain, a high Cu and Au content correlated with a low GSH content.

Since the glutathione content of the cells may also influence the metabolism of other metals than Cu, the overall metal composition was determined in all used strains cultivated with and without Au or Cu in a total of 780 measurements. The magnesium content, 11.8 ± 3.4 million Mg per cell in unchallenged cells of the parent strain AE104, was more than twofold (*D* >1) increased in the mean values of the biological repeats of 39 strains and growth conditions (Table S2, only *Q* >2 listed), in 10 of these more than threefold (Table S2, bold). The addition of Au or Cu did not influence the magnesium content nor was it affected by a ∆*gshA* deletion or a complementation of such a ∆*gshA* strain with *gshA* in *trans*. The strains with an increased magnesium content were all triple- and the quadruple-deletion strains in copper resistance genes, namely ∆*cop ∆cup ∆cus, ∆cop ∆cup ∆gig, ∆cop ∆cus ∆gig,* ∆*cup ∆cus ∆gig,* or ∆*cop ∆cup ∆cus ∆gig*. As in case of other strains with a disturbed metal homeostasis (20–22), a messed-up copper homeostasis also results in a higher cellular magnesium content.

While the magnesium content was up-regulated in many strains, the iron content was not affected by deletion of copper efflux systems in the presence or absence of Au or Cu. Exceptions were four strains, which displayed an iron content that was less than half of that of the parent AE104 cultivated without additions (787,000 ± 265,000 Fe per cell). These were all cultivated in the presence of Au and Cu and all ∆*gshA* mutants of the triple mutants ∆*cop ∆cus ∆gig, ∆cop ∆cup ∆gig, ∆cop ∆cup ∆cus* and the quadruple mutant ∆*cop ∆cup ∆cus ∆gig* (Table S3). Iron homeostasis or its acquisition was only disturbed in ∆*cop* mutants with at least two other deletions in copper resistance systems, plus deletion of ∆*gshA,* and addition of Au and of Cu ions to the cells. Only a combination of many damaging factors influences iron homeostasis. In contrast to iron was the cellular zinc level not affected by deletions in the copper resistance genes in the presence of Au and remained at 69,300 ± 10,400 Zn per cell.

### Metal resistance of the ∆*gshA* mutant strains

Resistance to copper further decreased in the presence of Au in all ∆*gshA* mutants of strain AE104 and in all single and double mutants, except ∆*cup ∆gig* and ∆*cup ∆cus* (Table 4). In these two double mutants, and in the related ∆*cup ∆cus ∆gig* triple mutant, Au complexes increased the copper resistance level, as in the strains with the wild-type *gshA* gene. These were the strains with an active Cop system and copper resistance on a very low level. As a possible explanation, binding of Au(I) to CopA instead of Cu(I) may not completely abolish the activity of the enzyme but allow a low residual activity sufficient to mediate this very low copper resistance level.

While in nearly all strains, copper resistance decreased with the ∆*gshA* deletion, as well in the absence and in the presence of Au (Table 4, two rows at the left hand), this was not the case in the *∆cup ∆cus* mutant but was observed for the *∆cup ∆cus* ∆*gig* mutant. This indicated that GSH was needed not only for copper resistance, as published (10), but also to resist synergistic Au-Cu toxicity. The exception was when Cus and Cup were absent and a residual copper resistance was mediated by Cop and Gig.

The ∆*gshA* mutant of the quadruple-deletion strain, and of the two triple-deletion strains that possessed Gig only or Cus only, was on the lowest IC_50_ level between 0.5 and 0.6 µM Cu when grown in either the presence or absence of Au ions. A synergistic toxicity of Cu and Au could no longer be observed at this low resistance level. As published (10), Gig and Cus alone were not able to mediate any degree of copper resistance. Adding the ability to synthesize GSH in these three mutants increased copper resistance by about threefold. The Cus system could be activated by Au complexes. Adding Cop to the ∆*gshA* mutant of the quadruple strain did not significantly increase resistance to copper in the absence of Au so that Cop appears unable to function alone. The same result had also been published for copper resistance (10). Finally, adding Cup increased copper resistance nearly 40-fold and, together with GSH, another 8-fold, and synergistic Cu Au toxicity was also restored.

## DISCUSSION

### New insights into resistance to synergistic gold-copper toxicity

This publication presents a new insight into the synergistic Au-Cu toxicity. The experiments with the self-made Au(III) solution had revealed a synergistic toxicity of Cu and Au that was based on a disturbed cytoplasmic copper homeostasis. The new data indicated a second layer of synergistic Cu-Au toxicity caused by the commercial Au(III) solution by an additional inhibition of the periplasmic Cu homeostasis. Evidence was also obtained that the MerR-type regulator CupR could be the master regulator for copper homeostasis in *C. metallidurans* and that glutathione was involved in many processes concerning the interaction of this bacterium with Cu and Au species.

The commercial Au(III) solution seems to generate a higher concentration of Au(I) in the periplasm of *C. metallidurans* than the previously used, self-made one. The latter one was responsible for the first layer of the synergistic Au/Cu toxicity due to an inhibition of the Cu(I)-exporting P_IB1_-type ATPase, CupA, which explained the increased accumulation of Cu atoms in the presence of Au atoms (8). The periplasmic Cu(I)/Au(I) oxidase could decrease the periplasmic Au(I) and Cu(I) concentration further, resulting in a decreased accumulation of Au and Cu atoms. This process occurred most efficiently in cells pre-incubated in the presence of Cu ions, which up-regulated *cop* (9). The cells also accumulated more Au in the presence of Cu than without Cu (Table 2) because both monovalent cations presumably compete for CopA.

Nevertheless, CopA was able to protect the cells against the previously used Au(III) solution sufficiently to allow the formation of Au nanoparticles (8). The commercially available Au(III) solution was five times more toxic than the previously used one. Interestingly, resistance to it increased when copper resistance systems were deleted, especially those that mediated copper resistance by removing periplasmic Cu(I) ions. This indicated that the commercial Au(III) solution interfered with the periplasmic Cu(I) homeostasis, resulting in inhibition of metalation of periplasmic Cu(I)-binding sites, for instance, of the Cu-Zn superoxide dismutase or the terminal oxidase of the respiratory chain (23–27) but also of CopA. This also prevented the re-oxidation of periplasmic Au(I) to Au(III), which subsequently enhanced the toxicity of periplasmic Au(I) and its import into the cytoplasm. Consequently, the commercial Au(III) solution was so toxic that the cells had no time to produce Au nanoparticles. Alternatively, lack of ability to produce the nanoparticles led to an even higher toxicity of this solution. The combination of all these processes generated a second layer of synergistic Au-copper toxicity based upon periplasmic processes.

The newly observed feature of the commercially available Au(III) solution argues for a higher generation rate of periplasmic Au(I) by this solution compared to the previously used one. This resulted in an increased accumulation of Au atoms by the cell and impediment of periplasmic Cu(I) homeostasis. Consequently, Au(III) was five times more toxic when additionally essential Cu(I) was removed from the periplasm and about two times more when this was not the case (Table 1).

An effect of the periplasmic, Cu-dependent Cu(I)/Au(I) oxidase CopA on the accumulation of Au and Cu was not evident at low Cu concentrations. CopA may be ineffective because of the inhibited Cu(I) homeostasis in the presence of the new Au solution. CopA is exported to the periplasm by the twin-arginine transport system. The CopA-ortholog CueO from *E. coli* is a transporter in an incompletely folded form (28). In *C. metallidurans,* the factors CopCD, which are not present in *E. coli*, may have imported Cu ions, or Cu(II), which is imported via a different pathway, might be reduced by GSH to provide the required Cu(I) for initial loading of the cofactor to CopA before export in a partially folded and metalated form (10, 29, 30). Full loading may require periplasmic Cu(I) ions. In the presence of Au and Cu, the Cop system was clearly required to decrease the cellular Cu content in the presence of Au (Table 3). More periplasmic Cu(I) was available under these conditions to overcome the Au-mediated inhibition of periplasmic Cu(I) homeostasis, providing sufficient cofactor to CopA.

The Cus system, which contained the transenvelope efflux pump CusCBA and the periplasmic copper chaperones CusF and CusD, contributed to copper resistance due to removal of periplasmic Cu(I) ions, which would otherwise cause toxicity in the periplasm and would be imported into the cytoplasm. Cus competed with Cop for periplasmic Cu(I) ions and cooperated with the inner membrane efflux pump CupA, which removed cytoplasmic Cu(I) ions to the periplasm for oxidation by CopA or further export by CusCBA (10). Cus contributed to Au toxicity because it removed essential Cu(I) from the periplasmic Cu(I) pool, which increased the inhibitory effect of the commercial Au solution on periplasmic Cu(I) homeostasis. No evidence was found for an export of periplasmic Au(I), as might be the case for the related GesCBA system from *Salmonella* (31). Expression of *cus* was induced by the commercially available Au solution and enhanced in a ∆*cop* mutation, indicating that the Cop system decreased the availability of the *cus* inducer (Fig. 4). In contrast to the up-regulation of *cus* by periplasmic Cu ions, up-regulation in response to Au complexes was *gig* dependent. However, the Gig system was only involved in the enhanced up-regulation of *cus* by Au in the ∆*cop* strain but not in any other strains. This makes sense only if two periplasmic Au species are assumed to serve as *cus* inducers. One that is stabilized by the action of Gig and efficiently removed by Cop, and a second that is not affected by these two systems. The membrane-integral GigT in combination with the periplasmic GigP and oxidized GSSG may oxidize periplasmic Cu(I) to Cu(II) by feeding the electron into the quinol pool of the respiratory chain (10) so that the two inducing Au species could be a periplasmic Au-glutathione complex Au-SG and an Au species not bound to GSH.

Surprisingly, the Cup determinant was also involved in the up-regulation of *cus* by Au. There was no evidence that the P_IB1_-type Cu(I) efflux system, CupA, exported cytoplasmic Au(I). Instead, it was inhibited by Au (7, 8). The other two products of the *cup* determinant were the copper chaperone CupC and the MerR-type regulator CupR, which both bind Au (11, 12). There is no two-component regulatory system in the vicinity of the *cus* determinant, as there is in *E. coli* (18, 32–34), although *cus* induction by Cu is clearly under the control of periplasmic Cu ions (10). The CopR_2_S_2_ two-component regulatory system was involved in Cu-dependent up-regulation of *cus* but not essential for this process. This indicates that a complicated regulatory network composed of CupR and the CopR_2_S_2_ regulatory system may be in control of the copper resistance determinants in the plasmid-free *C. metallidurans* strain AE104.

Expression of *gig* is controlled by periplasmic Cu ions (10). Commercially available Au solution also resulted in an up-regulation of *gig,* and this process was glutathione dependent, meaning that the *gig* inducer was derived from Au complexes and GSH, for instance, periplasmic Au-SG, as in the case of *cus* regulation. Directly adjacent to *gig* are the genes for a sigma factor of the extracytoplasmic function (ECF) family (35, 36) and RpoQ along with its anti-sigma factor RsqA (20, 37, 38). Unfortunately, regulation of *gig* by RpoQ has not been demonstrated yet because any deletion of *rpoQ* was complemented partially by other sigma factors in *C. metallidurans*. RpoQ and its paralog RpoR are involved in thiol homeostasis of *C. metallidurans* (20), which agrees with the interaction of GigPT with oxidized periplasmic GSSG and electron transfer to and from the quinol pool. This indicates that an Au-glutathione complex may be the *gig* inducer via RsqA and RpoQ so that this ECF sigma factor may sense periplasmic metal-GSH complexes.

GSH can be exported into the periplasm by a CydDC-type bacterial glutathione exporter (39). Rmet_0391 or AtmA (10), which is involved in nickel tolerance in *C. metallidurans* (40), is highly related (29% identity in 428 aa) to CydD. The related Atm1 from another proteobacterium was involved in export of transition metal-GSH complexes, mediating mercury and silver resistance (41–43), making AtmA a candidate for an Au-SG efflux system.

When *C. metallidurans* comes into contact with 50 µM of the previously used Au(III) solution, Au was imported into the cytoplasm within minutes and present as Au(I)-S species within a couple of hours. Subsequently and within 72 h, half of the Au was still present as Au(I)-S species, but 29% was metallic Au, and Au “hot spots” were associated with the cell envelope (6). Slow removal of Au-SH to the periplasm by AtmA would explain this process.

There was also a decrease in the cellular glutathione content under two conditions. First, in all ∆*cus* cells, and even more in the presence of Au from the commercially available Au solution (Table 5). Second, the level of GSH was decreased in the presence of Au and Cu in the ∆*cop ∆cup* and the ∆*cop ∆cus* double mutants, all triple, and the quadruple mutants (Table 5). Absence of the ability to remove periplasmic Cu(I) by Cus or a disturbed periplasmic copper homeostasis combined with a high Cu availability resulted in a decreased cellular glutathione content.

A diminished GSH content may be the result of decreased GSH synthesis, increased degradation, or increased export, for instance, of metal-SG complexes by AtmA. Cytoplasmic metal-SG complexes might be degraded, and as shown with cadmium, soft heavy metal ions may inhibit thiol synthesis (44, 45). Absence of the Cus system with the CusCBA transenvelope efflux system and the periplasmic Cu(I) chaperones CusF and CusD should disturb periplasmic Cu(I) homeostasis, which could be compensated by export of GSH or cytoplasmic Cu(I)-SG by AtmA. Likewise, absence of Cop in the presence of Au and Cu was associated with a decreased GSH content and a higher accumulated level of Cu (Tables 3 and 5) so that export of Cu-SG by AtmA might be a mechanism to relieve the high cellular Cu and Au content. However, this process is very slow. With the previously used Au solution, the cells contained only 50,000 Au per cell (Table 2), and export of approximately 50% of them required 72 h (6), which calculated to 5.44 exported Au per minute and cell. It might be easier to study this export reaction in cells containing a million Au per cell due to incubation with the commercially available Au(III) solution.

### The procedure used to produce Au(III) solutions has biological consequences

Formation of nanoparticles from the cations of the noble metals Cu, Ag, and Au is a chemical process, depending on thermodynamics and kinetics. Since only exergonic reactions are possible, the reduction of a cation to the metallic form requires a positive redox potential. Using the Nernst equation, this can be derived from the standard redox potentials of the halve cells and the concentrations of the substrates and products (46). The energy of the transition state for the redox reaction determines the reaction rate of the subsequent reaction, the kinetics of this reaction. The standard redox potentials of the noble metal cations increase from Cu to Au due to increased shielding of the valence electrons from the atomic nucleus by the increasing number of filled inner orbitals (Table 6). The standard potentials for the formation of metallic copper from the Cu(I) and Cu(II) cations are close to that of glutathione. GSH keeps cytoplasmic thiol groups reduced, which allows the formation of thioesters as second major energy-rich bound of cellular biochemistry, besides acid anhydrides (47). Cytoplasmic Cu(II) should be immediately bind to GSH, yielding rapidly Cu(I)-GSH_2_ and also Cu(II)-GSSG, which can be reduced back to Cu(I)-GSH_2_ by the high cytoplasmic concentration of GSH inside the cell (45, 48, 49). The strong bond to the sulfur atom protects against further reduction of Cu to the metallic form in the cytoplasm of a living cell, but cellular extracts are able to offer sufficient redox power for the formation of Cu nanoparticles (50).

**TABLE 6:** Redox potential of noble metal ions^*a*^

Oxidized form	Reduced form	Eo*'* (mV)
Ag_2_S	Ag^0^	−1,111
Cu_2_O	Cu^0^	−780
Cu(OH)_2_	Cu^0^	−642
2 H^+^	H_2_	−421
NAD^+^	NADH	−320
Cu^2+^	Cu^+^	−267
**GSH**	**GSSG**	**−240**
AgCl	Ag^0^	−198
Cu^2+^	Cu^0^	−70
Ubiquinone	Ubiquinol	100
Cu^+^	Cu^0^	101
2 Cyt *c* ox	2 Cyt *c* red	235
Ag^+^	Ag^0^	380
AuCl_4_^−^	Au^0^	582
O_2_/2	H_2_O	816
Au^3+^	Au^+^	981
Au(OH)_3_	Au^0^	1,030
Au^3+^	Au^0^	1,078
Au^+^	Au^0^	1,272

^*a*^ The redox potential Eo′ at pH = 7 for the metal cations was calculated from the standard halve cell potentials Eo at pH = 0 (51). For the organic compounds, the sources already listed Eo′ (52, 53). The shaded field list redox reaction outside of the physiological range, which is between the redox potential of protons to molecular hydrogen and of molecular oxygen to water. The potential of glutathione was bold faced to highlight it.

The standard redox potential of Ag is well within the physiological range (Table 6), and Ag nanoparticles are formed by bacteria, fungi, and plant extracts (54). The Ag(I) cation is very sensitive against light and forms complexes with very low solubility with chloride and sulfide, which also affects the standard potential (Table 6). Nitrate reductases and hydrogenases may catalyze formation of Ag nanoparticles (55). The standard potential of Au(I) and Au(III) is the most positive one among the noble metal cations. Except Au(III)Cl_4_^−^, which has a standard potential less positive than that of water, Au ions should be able to oxidize water (Table 6). Only kinetic barriers, for instance, binding to a thiol group of Cl^−^ anions, keep Au ions in a meta-stable condition. Especially for Au(III)Cl_4_^−^ with its comparable low potential among the Au ions is the mode of contact with the reduced compounds of the cellular biochemistry, a decisive factor for the possibility of the subsequent reduction, and the way how it occurs.

Thermodynamic calculations that include solvation and relativistic effects indicate that Au(III)Cl_4_^−^ undergoes a partial hydrolysis to AuCl_3_OH^−^, which is stable toward Cl_2_ elimination that would generate Au(I)Cl_2_^−^ (56). In contrast to AuCl_3_OH^−^, Au(I)Cl_2_^−^ would undergo a disproportionation to Au(III)Cl_4_^−^, Au(0)_s_, and 2 Cl^−^. This reaction is exergonic due to the cohesion energy of solid Au (56). AuCl_3_OH^−^ may undergo further hydrolysis to Au(III)Cl_2_OH_2_^−^, and both species should be present at equal concentrations at neutral pH (57). Under additionally reducing conditions, for instance, in the vicinity of living cells, low-order Au(I) complexes should become predominant (58). Consequently and in analogy to Cu(II) (59), AuCl_3_OH^−^, which is stable in solution, should be reduced within the periplasm of *C. metallidurans* upon contact with its respiratory chain components to Au(I) complexes. These would undergo exergonic disproportionation back to Au(III) complexes and metallic Au as nanoparticles in the periplasm of *C. metallidurans*.

Moreover, like Cu(I), Au(I) species may be more rapidly transported into the cytoplasm of *C. metallidurans* than the more oxidized species Au(III) and Cu(II). This is demonstrated by the different IC_50_ values of Cu(II) and Cu(I) observed for *Escherichia coli* of about 1,500 µM Cu(II) compared to about 5 µM Cu(I) (60). Na(I)- or K(I)-dependent import systems (33, 60) could be responsible for this effect. Inside the cytoplasm, Au(I) inhibits the Cu(I)-exporting P_IB1_-type ATPase, CupA, so that Au and Cu ions exert synergistic toxicity (8) because Cu(I) can no longer be removed from this compartment. *C. metallidurans* is able to resist this synergistic toxicity by oxidation of periplasmic Au(I) back to Au(III) species through the activity of the Cu-dependent oxidase, CopA (Fig. 1), which decreases the accumulation of Au atoms in the cells (9).

The previously used Au(III) solution had been prepared using *aqua regia* to oxidize metallic Au. The product was suspended in distilled water to a concentration of 50 mM (6). A straightforward and cost-effective route to an Au(III) solution from metallic Au uses chlorine gas (61). In this solution, Au(III)Cl_4_^−^ is stable over at least a year due to the residual presence of Cl_2_ but may slowly and partially convert to Au(I)Cl_2_^−^ due to the escape of the chlorine gas in the periplasm of *C. metallidurans* during its growth with shaking, in addition to the reduction upon contact with the respiratory chain components. Subsequent loss of a Cl^−^ would lead to Au(I)Cl. The electronegativity in the Pauling scale of Au is 2.4 and Cl is 3.0 (62), leading to a difference in electronegativity of Au-Cl of 0.6, in other words, with an ionic character of the bond of 9%. The Au-Cl bond is nearly covalent, which is also emphasized by the weak charge separation in AuCl_2_^−^ (56). The electronegativity of Hg is 1.9, the difference to that of Cl 1.1, and this bond can be considered to 26% an ionic bond. Despite this partial ionic character, HgCl_2_ is able to diffuse across biological membranes (63). Diffusion is slow so that mercury resistance mechanisms involve active transport across the cytoplasmic membrane by proteins such as MerT, followed by reduction to metallic, volatile mercury by MerA (64). It can be expected from this comparison that AuCl with its weak charge separation diffuses more rapidly across biological membranes than HgCl_2_.

The previously used Au(III)Cl_4_^−^ solution would be present as Au(III)Cl_3_OH^−^ in the periplasm of *C. metallidurans* and would require contact with the respiratory chain to be reduced to Au(I) species, which are transported into the cytoplasm to exert toxic effects and may disproportionate leading to Au nanoparticles (6, 7). In contrast, a commercially available Au(III)Cl_4_^-^ solution prepared by the chlorine solution method would lead more rapidly to Au(I)Cl_2_^−^ and AuCl in the periplasm and exert toxic effects already in this compartment by inhibition of metalation of Cu(I)-dependent proteins. Consequently, all resistance systems that removed periplasmic Cu(I) such as CusCBA, CopA, and Gig decreased Au resistance (Table 1) because removal of periplasmic Cu(I) enhanced the Au-mediated metalation of metal-binding sites by periplasmic Cu(I). Moreover, Au ions may be imported into the cytoplasm rapidly and in part by diffusion of AuCl across the membrane, which results in a 20-fold higher Au content of the cells, as has been observed (Tables 2 and 3). Together, both effects resulted in a five times lower Au resistance in the parental strain AE104 in the case of the commercially available compared with the previously used (6–9) Au solution.

### Conclusion

Two differently prepared Au(III) solutions had different effects on *C. metallidurans* and its copper resistance (Fig. 1). The solution prepared using *aqua regia* may be predominantly present as Au(III)Cl_3_OH^−^, stable against the release of chlorine, which leads to formation of Au(I). Reduction requires contact with the electrons of the respiratory chain of *C. metallidurans,* and the Cu-dependent Au(I) oxidase CopA could keep the level of periplasmic Au(I) low. This would result in a relatively low accumulation of Au by the cells and ability to form periplasmic Au nanoparticles, which permanently remove the toxic Au cations from the solution. On the other hand, chlorine-prepared Au(III) may remain stable as Au(III)Cl_4_^−^ and could react to Au(I)Cl_2_^−^ and AuCl, leading to the enhanced accumulation of Au by the cells and inhibition of the periplasmic Cu(I) homeostasis system, which are also needed to supply Cu ions to CopA. This adds a second layer to the synergistic Au-Cu toxicity in this bacterium. The cells may try to remove this high load of Au by export of Au-glutathione complexes in a very slow process. Since the speciation of Au species in auriferous soils may vary (14, 57, 65–67), the use of a different Au solution has opened a route to understand the complicated interaction of a bacterium with Cu and Au in the environment.

## MATERIALS AND METHODS

### Bacterial strains and growth conditions

Strains used for experiments were derivatives of the plasmid-free derivative AE104 of *C. metallidurans* CH34 (3) and are listed in Table S4. These strains were also described in a previous publication (10). Additionally, vector pBBR (68) and the same vector carrying the complete *gshA* gene were transferred into these strains. Tris-buffered mineral salts medium (3) containing 2 g sodium gluconate/L (TMM) was used to cultivate these strains aerobically with shaking at 30°C. The medium had been improved compared to the previously published medium by choosing mineral salts with a higher purity. Solid Tris-buffered media contained 20 g agar/L. Strains were routinely transferred to fresh TMM plates every 2 weeks and taken from the −80°C stock culture twice a year. HAu(III)Cl_4_ was provided by Sigma-Aldrich (TraceCERT , 1 g/L Au in hydrochloric acid)

### Dose-response growth curves in 96-well plates

Experiments were conducted in TMM. A pre-culture was incubated at 30°C, 200 rpm up to early stationary phase, then diluted 1:20 into fresh medium, and incubated for 24 h at 30°C and 200 rpm. Overnight cultures were used to inoculate parallel cultures with increasing metal concentrations in 96-well plates (Greiner). Cells were cultivated for 20 h at 30°C and 1,300 rpm in a neoLab Shaker DTS-2 (neoLab, Heidelberg, Germany), and the optical density was determined at 600 nm in a TECAN infinite 200 PRO reader (Tecan Group Ltd., Männedorf, Switzerland) as indicated. To calculate the IC_50_ values (inhibitory concentration: metal concentration that led to turbidity reduction by half) and the corresponding *b*-value (measure of the slope of the sigmoidal dose-response curve), the data were adapted to the formula OD(*c*) = OD0/{1 + exp[(*c* - IC_50_)/*b*]}, which is a simplified version of a Hill-type equation as introduced by Pace and Scholtz (69) as published (70). OD(*c*) is the turbidity at a given metal concentration, OD0 that had no added metal, and *c* the metal concentration.

### β-Galactosidase assay

*C. metallidurans* cells with a *lacZ* reporter gene fusion (10) were cultivated as a pre-culture in TMM containing 1.5 g L^−1^ kanamycin at 30°C, 200 rpm for 18 h, diluted 20-fold into fresh medium with 1 g L^−1^ kanamycin, incubated with shaking at 30°C for 24 h, diluted 66-fold into fresh medium, and incubated with shaking at 30°C until a cell density of 100 Klett units was reached. This culture was distributed into sterile 96-well plates (Greiner Bio-One, Frickenhausen, Germany). After addition of metal salts, incubation in the 96-well plates was continued for 3 h at 30°C in a neoLab Shaker DTS-2 (neoLab Migge Laborbedarf, Heidelberg, Germany). The turbidity at 600 nm was determined using a TECAN Infinite 200 Pro reader (TECAN, Männedorf, Switzerland), and the cells were sedimented by centrifugation at 4°C for 30 min at 4,500 × *g*. The supernatant was discarded, and the cell pellets were frozen at −20°C. For the enzyme assay, the pellet was suspended in 190 µL Z buffer (60 mM Na_2_HPO_4_, 40 mM NaH_2_PO_4_, 10 mM KCl, 1 mM MgSO_4_, 0.05 M beta-mercaptoethanol), and 10 µL permeabilization buffer was added (6.9 mM CTAB, cetyl-trimethyl-ammonium bromide, 12 mM sodium deoxycholate). The suspension was incubated with shaking at 30°C, and 20 µL ONPG solution (13.3 mM ortho-nitrophenyl-beta-D-galactopyranoside in Z-buffer without beta-mercaptoethanol) was added. Incubation was continued with shaking in a neoLab Shaker DTS-2 at 30°C until the yellow color of *o*-nitrophenol was clearly visible, and the reaction was stopped by addition of 50 µL 1 M Na_2_CO_3_. The extinction at 420 and 550 nm was measured in a TECAN Infinite 200 Pro reader. The activity was determined as published (71) with a factor of 315.8 µM calculated from the path length of the 96-well plate and the extinction coefficient of *o*-nitrophenol:


$$activity=315.8\mu M\;*\;\left\{E_{420}-(1.75\;*\;E_{550})\right\}\;/\;reaction\;time$$

specific activity: activity divided by the cellular dry mass (71).

### Genetic techniques

Standard molecular genetic techniques were used (72, 73). For conjugative gene transfer, overnight cultures of donor strain *E. coli* S17/1 (74) and of the *C. metallidurans* recipient strains grown at 30°C in Tris- buffered medium were mixed (1:1) and plated onto nutrient broth agar. After 2 days, the bacteria were suspended in TMM, diluted, and plated onto selective media as previously described (72). Primer sequences are provided in Table S5.

### Gene deletions

Primer sequences are also provided in Table S5. Plasmid pECD1002, a derivate of plasmid pCM184 (75), was used to construct deletion mutants. These plasmids harbor a kanamycin resistance cassette flanked by *loxP* recognition sites. Plasmid pECD1002 additionally carries alterations of 5 bp at each *loxP* site. Using these mutant *lox* sequences, multiple gene deletions within the same genome are possible without interferences by secondary recombination events (76, 77). Fragments of 300 bp upstream and downstream of the target gene were amplified by PCR, cloned into vector pGEM T-Easy (Promega), sequenced, and further cloned into plasmid pECD1002. The resulting plasmids were used in a double-crossover recombination in *C. metallidurans* strains to replace the respective target gene by the kanamycin resistance cassette, which was subsequently also deleted by transient introduction of *cre* expression plasmid pCM157 (75). Cre recombinase is a site-specific recombinase from the phage P1 that catalyzes the *in vivo* excision of the kanamycin resistance cassette at the *loxP* recognition sites. The correct deletions of the respective transporter genes were verified by southern DNA-DNA hybridization. For construction of multiple deletion strains, these steps were repeated. The resulting mutants carried a small open reading frame instead of the wild-type gene to prevent polar effects.

### Gene insertions and disruptions

For reporter operon fusions, *lacZ* was inserted downstream of several targets. This was done without interrupting any open reading frame downstream of the target genes to prevent polar effects. The 300–400 bp 3′ ends of the respective target genes were amplified by PCR from total DNA of strain AE104 and the resulting fragments cloned into plasmid pECD794 (pLO2-*lacZ*) (78). The respective operon fusion vectors (pECD1386 for *cusF-lacZ*, pECD1667 for *gigT-lacZ*) were inserted into the open reading frame of the target gene by single crossover recombination. Using the 300 bp 5′ part of the *gshA* gene, this procedure was also used to interrupt the *gshA* gene with pECD1668 in strain AE104 parent strain and its mutant derivatives.

### Inductively coupled plasma mass spectrometry

Cells were incubated in TMM for 20 h at 30°C with shaking at 200 rpm, diluted 20-fold into fresh TMM medium, and shaking was continued at 30°C for 24 h. Cells were diluted 66-fold into fresh medium until 100 Klett were reached (mid-exponential phase of growth). Metals were added, and the cells were left growing until they reached 150 Klett. Ten milliliters of the cells were harvested by centrifugation, washed twice with 50 mM TrisHCl buffer (pH 7.0) containing 10 mM EDTA and 150 mM NaCl at 4°C. For ICP-MS analysis, 420 µL *aqua regia* (nitric acid and hydrochloric acid, ratio 1:3, analytical grade) was added to the samples, and the mixture mineralized at 70°C overnight. Samples were diluted to a final concentration of 5% (vol/vol) hydrochloric acid and 3% (vol/vol) nitric acid. Indium and germanium were added as internal standards at a final concentration of 10 ppb each. Elemental analysis was performed via ICP-MS using Cetac ASX-560 sampler (Teledyne, Cetac Technologies, Omaha, Nebraska), a MicroFlow PFA-200 nebulizer (Elemental Scientific, Mainz, Germany), and an ICAP-TQ ICP-MS instrument [Thermo Fisher Scientific, Bremen) operating with a collision cell and flow rates of 4.8 mL × min^−1^ of He/H_2_ [93%/7% (79)], with an Ar carrier flow rate of 0.76 L × min^−1^ and an Ar make-up flow rate at 15 L × min^−1^. An external calibration curve was recorded with ICP-multi-element standard solution XVI (Merck) in 5% (vol/vol) hydrochloric acid. The sample was introduced via a peristaltic pump and analyzed for its metal content. For blank measurement and quality/quantity thresholds, calculations based on DIN32645 TMM were used. The results were calculated from the ppb data as atoms per cell as described (80).

### Glutathione determination

Cells were cultivated as described above for ICP-MS. And, 5 mg cells were harvested (30 min, 4,500 × *g*, 4°C) and washed twice with TMM. Cell pellets were resuspended in 100 µL 5% SSA (5-sulfosalicylic acid) solution and disrupted by three freeze-thawing cycles (liquid nitrogen, water bath at 37°C, 2 min per treatment, three repeats). Cell debris was removed by centrifugation (30 min, 4,500 × *g,* 4°C). The supernatant was used to determine the protein concentration with the QuantiPro BCA Assay Kit (Sigma-Aldrich, Taufkirchen, Germany) using bovine serum albumin as a standard and to measure the GSH content by the Glutathione Assay Kit (CS0260, Sigma-Aldrich, Taufkirchen, Germany) according to the manufacturer’s instructions. The enzymatic determination of the total amount of glutathione (GSH and GSSG) after deproteinization with SSA was measured photometrically at 412 nm by increasing amounts of TNB using a kinetic assay.

### Transmission electron microscopy

A pre-culture was incubated at 30°C, 200 rpm to early stationary phase, diluted 1:20 in fresh medium, and incubated for 24 h at 30°C and 200 rpm. Cells were diluted 66-fold into fresh TMM until they reached 180–200 Klett. Au(III)-chloride (prepared using *aqua regia* or commercially available from Sigma-Aldrich) was added at a final concentration of 50 µM, and cells were incubated with shaking at 100 rpm for 72 h at 30°C. Alternatively, cells were incubated with commercially available Au(III)-chloride for time intervals between 15 min and 48 h and with concentrations between 15 and 50 µM, harvested at 4,500 g at 4°C, washed once in TMM, and then resuspended in fresh Au-free TMM. Cells were allowed to recover during incubation with shaking at 100 rpm and at 30°C for up to 72 h.

Cells were fixed directly with 3% glutaraldehyde (Sigma, Taufkirchen, Germany) in 0.1 M sodium cacodylate buffer (SCB) for 3 h, centrifuged at 5,000 rpm for 5 min, and suspended in 4% agar/SCB, followed by one wash-step with SCB-buffer overnight at 4°C and three wash-steps with the same buffer for 5 min. After post-fixation with osmium tetroxide for 1 h, samples were dehydrated in a series of ethanol (10%, 30%, and 50%). Then cells were treated with 1% uranylacetate/70% ethanol for 1 h and further dehydrated with a series of 70%, 90%, and 100% ethanol. Thereafter, the samples were infiltrated with epoxy resin according to reference (81) and polymerized at 70°C. The ultrathin sections (80 nm) were analyzed using an EM900 transmission electron microscope (Carl Zeiss SMT, Oberkochen, Germany) operating at 80 kV. The images were recorded with a Variospeed SSCCD camera SM-1k-120 (TRS, Moorenweis, Germany).

### Statistics

Student’s *t*-test was used, but in most cases, the distance (*D*) value, *D*, has been used several times previously for such analyses (7, 22, 82). It is a simple, more useful value than Student’s *t*-test because non-intersecting deviation bars of two values (*D* >1) for three repeats always means a statistically relevant (≥ 95%) difference, provided the deviations are within a similar range. At *n* = 4, significance is ≥97.5%, at *n* = 5 ≥99% (significant) and at *n* = 8 ≥99.9% (highly significant).

## SUPPLEMENTAL MATERIAL

Supplemental material aem.00146-24-s0001.pdf Tables S1 to S5; Figures S1 to S4.

ASM does not own the copyrights to Supplemental Material that may be linked to, or accessed through, an article. The authors have granted ASM a non-exclusive, world-wide license to publish the Supplemental Material files. Please contact the corresponding author directly for reuse.
