# Supplementary material for: A gold speciation that adds a second layer to synergistic gold-copper toxicity in Cupriavidus metallidurans
Source: Appl Environ Microbiol. 2024 Apr 1;90(4):e00146-24. doi: 10.1128/aem.00146-24 (PMC11022561; doi:10.1128/aem.00146-24)
Supplement: Supplemental material — Tables S1 to S5; Figures S1 to S4. [file aem.00146-24-s0001.pdf]

Supplementary Tables and Figures

Supplementary Table S1. Gold and copper content of the mutant cells[a].

Strain	2.5 μM Au<br>Au (1000/cell)	2.5 μM Au + 10 μM Cu<br>Au (1000/cell)	Cu (1000/cell)	Q(AuCu_Au)
AE104	961±264; 1.0	1476±296; 1.0	82±27; 1.0	1.54
ΔgshA (marker-free)	1319±148; 1.4	1870±279; 1.3	75±7; 0.9	1.42
ΔgshA (pBBR)	1392±113; 1.4	1938±413; 1.3	80±6; 1.0	1.39
ΔgshA (pBBR::*gshA*)	1093±146; 1.1	1474±190; 1.0	90±26; 1.1	1.35
Δ*gshA* (disrupted)	1028±228; 1.1	**913±98; 0.6**	**40±5; 0.5**	0.89
Δ*gshA* (pBBR::*gshA*)	1196±245; 1.2	1316±138; 0.9	92±15; 1.1	1.10
AE104(pBBR)	1207±289; 1.3	2447±2384; 1.7	134±124; 1.6	2.03
AE104(pBBR::*gshA*)	1261±357; 1.3	1869±311; 1.3	105±23; 1.3	1.48
Δcop	1003±211; 1.0	1678±268; 1.1	**280±112; 3.4**	**1.67**
Δcop Δ*gshA*	1087±190; 1.1	1184±62; 0.8	**205±27; 2.5**	1.09
Δcop Δ*gshA*(pBBR)	970±58; 1.0	2068±975; 1.4	374±327; 4.6	**2.13**
Δcop Δ*gshA*(pBBR::*gshA*)	1098±391; 1.1	1152±232; 0.8	**234±105; 2.8**	1.05
Δcop Δcup	980±417; 1.0	1599±225; 1.1	**309±89; 3.8**	1.63
Δcop Δcup Δ*gshA*	828±385; 0.9	**894±105; 0.6**	**209±100; 2.5**	1.08
Δcop Δcup Δ*gshA*(pBBR)	983±89; 1.0	1363±227; 0.9	**248±56; 3.0**	1.39
Δcop Δcup Δ*gshA*(pBBR::*gshA*)	1321±143; 1.4	1191±226; 0.8	**314±57; 3.8**	0.90
Δcop Δcup Δcus	893±258; 0.9	1435±174; 1.0	**326±56; 4.0**	**1.61**
Δcop Δcup Δcus Δ*gshA*	910±146; 0.9	1147±96; 0.8	**366±42; 4.5**	1.26
Δcop Δcup Δcus Δ*gshA*(pBBR)	797±58; 0.8	**908±47; 0.6**	**262±18; 3.2**	1.14
Δcop Δcup Δcus Δ*gshA*(pBBR::*gshA*)	1349±214; 1.4	1481±308; 1.0	**351±43; 4.3**	1.10
Δcop Δcup Δcus Δgig	932±245; 1.0	1351±206; 0.9	**383±116; 4.7**	1.45
Δcop Δcup Δcus Δgig Δ*gshA*	911±180; 0.9	1325±21; 0.9	**416±124; 5.1**	1.46

Δcop Δcup Δcus Δgig ΔgshA(pBBR)	921±96; 1.0	1288±186; 0.9	**246±15; 3.0**	1.40
Δcop Δcup Δcus Δgig ΔgshA(pBBR::*gshA*)	1149±139; 1.2	1451±199; 1.0	**349±31; 4.2**	1.26
Δcop Δcup Δgig	925±255; 1.0	1454±237; 1.0	**272±50; 3.3**	**1.57**
Δcop Δcup Δgig ΔgshA	962±349; 1.0	**795±192; 0.5**	**139±29; 1.7**	0.83
Δcop Δcup Δgig ΔgshA(pBBR)	965±47; 1.0	1220±112; 0.8	**231±28; 2.8**	1.26
Δcop Δcup Δgig ΔgshA(pBBR::*gshA*)	**2008±210; 2.1**	**1636±153; 1.1**	**321±76; 3.9**	0.81
Δcop Δcus	1077±239; 1.1	1822±304; 1.2	**403±179; 4.9**	**1.69**
Δcop Δcus ΔgshA	1238±442; 1.3	1047±87; 0.7	**440±43; 5.4**	0.85
Δcop Δcus ΔgshA(pBBR)	927±58; 1.0	1498±291; 1.0	**331±218; 4.0**	**1.62**
Δcop Δcus ΔgshA(pBBR::*gshA*)	1348±80; 1.4	1626±299; 1.1	**574±181; 7.0**	1.21
Δcop Δcus Δgig	1079±213; 1.1	1653±169; 1.1	**280±52; 3.4**	**1.53**
Δcop Δcus Δgig ΔgshA	1238±441; 1.3	1102±266; 0.7	**366±83; 4.5**	0.89
Δcop Δcus Δgig ΔgshA(pBBR)	1451±282; 1.5	1987±161; 1.3	**268±34; 3.3**	1.37
Δcop Δcus Δgig ΔgshA(pBBR::*gshA*)	1172±110; 1.2	1335±204; 0.9	**366±183; 4.5**	1.14
Δcop Δgig	928±216; 1.0	1598±318; 1.1	**250±71; 3.0**	**1.72**
Δcop Δgig ΔgshA	1112±262; 1.2	1244±269; 0.8	**278±25; 3.4**	1.12
Δcop Δgig ΔgshA(pBBR)	959±127; 1.0	1432±236; 1.0	**188±52; 2.3**	1.49
Δcop Δgig ΔgshA(pBBR::*gshA*)	1210±245; 1.3	1412±212; 1.0	**246±63; 3.0**	1.17
Δcup	944±145; 1.0	1470±334; 1.0	99±37; 1.2	**1.56**
Δcup ΔgshA	993±265; 1.0	987±241; 0.7	**52±2; 0.6**	0.99
Δcup ΔgshA(pBBR)	1111±142; 1.2	1501±193; 1.0	79±22; 1.0	1.35
Δcup ΔgshA(pBBR::*gshA*)	1031±104; 1.1	1064±126; 0.7	102±25; 1.2	1.03
Δcup Δcus	941±225; 1.0	1514±357; 1.0	94±29; 1.1	1.61
Δcup Δcus ΔgshA	1205±135; 1.3	1130±275; 0.8	57±6; 0.7	0.94
Δcup Δcus ΔgshA(pBBR::*gshA*)	1043±98; 1.1	1310±108; 0.9	139±60; 1.7	1.26
Δcup Δcus Δgig	1021±216; 1.1	1459±384; 1.0	96±25; 1.2	1.43
Δcup Δcus Δgig ΔgshA	958±266; 1.0	999±98; 0.7	55±5; 0.7	1.04
Δcup Δcus Δgig ΔgshA(pBBR)	1094±150; 1.1	1312±100; 0.9	98±6; 1.2	1.20

Δcup Δcus Δgig ΔgshA(pBBR::*gshA*)	1328±114; 1.4	1649±297; 1.1	119±36; 1.5	1.24
Δcup Δgig	968±238; 1.0	1472±348; 1.0	100±34; 1.2	1.52
Δcup Δgig ΔgshA	1235±528; 1.3	1056±118; 0.7	55±11; 0.7	0.86
Δcup Δgig ΔgshA(pBBR)	1357±517; 1.4	1460±124; 1.0	73±10; 0.9	1.08
Δcup Δgig ΔgshA(pBBR::*gshA*)	1165±135; 1.2	1188±121; 0.8	119±19; 1.5	1.02
Δcus	741±208; 0.8	1188±199; 0.8	90±31; 1.1	**1.60**
Δcus ΔgshA	900±532; 0.9	**809±255; 0.5**	59±14; 0.7	0.90
Δgig	1013±276; 1.1	1542±314; 1.0	80±21; 1.0	1.52
Δgig ΔgshA	1029±284; 1.1	1173±216; 0.8	47±10; 0.6	1.14
Δgig ΔgshA(pBBR)	1075±112; 1.1	1449±51; 1.0	62±9; 0.8	1.35
Δgig ΔgshA(pBBR::*gshA*)	1095±136; 1.1	1374±610; 0.9	114±29; 1.4	1.25
Δgig Δcus	1049±211; 1.1	1530±349; 1.0	82±21; 1.0	1.46
Δgig Δcus ΔgshA	1269±333; 1.3	1302±527; 0.9	**43±4; 0.5**	1.03
Δgig Δcus ΔgshA(pBBR)	**1510±133; 1.6**	1679±254; 1.1	73±15; 0.9	1.11
Δgig Δcus ΔgshA(pBBR::*gshA*)	1014±156; 1.1	1239±166; 0.8	82±28; 1.0	1.22

[a]The metal content of the cells (in 1000 atoms per cell) was determined by ICP-MS in cells cultivated in the presence of 2.5 μM Au(III)HCl_3 and 2.5 μM Au(III)HCl_3 plus 10 μM Cu(II). The copper content is provided only for the Au/Cu-cultivated cells. The mean values of ≥ 3 determinations is provided with the deviation, and is followed by the ratio Q of this value with the AE104 result under the same conditions. Bold-faced letters indicate significant differences (D > 1, Q > 1.5 or Q < 0.67), green up-regulated and red down-regulated values. The last row compares the gold contents of the cells cultivated in the presence of Au(III) with and without added copper ions. The copper content of strain AE104 cultivated without added metals was 8,070±4,000 Cu per cell, the mean value of all cells cultivated without added metals or with 2.5 μM Au(III) was not different from this value and had a mean value of 7,230±2,200 Cu per cell. The orange field indicates a copper content down-regulated compared to the Δ*cop* strain, light green field an up-regulated value.

Supplementary Table S2. Bacterial strains with cellular magnesium contents different from strain AE104 parent[a]

Bacterial Strain	Addition	Q(AE104)	D(AE104)
Δcop Δcup Δcus	-	*0.89*	*0.35*
Δcop Δcup Δcus	2.5 µM Au	2.50	1.10
Δcop Δcup Δcus	2.5 µM Au, 10 µM Cu	2.69	1.86
Δcop Δcup Δcus Δgig	-	2.58	3.78
Δcop Δcup Δcus Δgig	2.5 µM Au	2.99	2.88
Δcop Δcup Δcus Δgig	2.5 µM Au, 10 µM Cu	2.56	1.61
Δcop Δcup Δcus Δgig ΔgshA	2.5 µM Au	2.25	2.58
Δcop Δcup Δcus Δgig ΔgshA(pBBR)	-	2.57	3.46
Δcop Δcup Δcus Δgig ΔgshA(pBBR)	2.5 µM Au	2.62	3.31
Δcop Δcup Δcus Δgig ΔgshA(pBBR)	2.5 µM Au, 10 µM Cu	2.47	3.35
Δcop Δcup Δcus Δgig ΔgshA(pBBR::gshA)	-	**3.61**	5.03
Δcop Δcup Δcus Δgig ΔgshA(pBBR::gshA)	2.5 µM Au	**4.01**	9.62
Δcop Δcup Δcus Δgig ΔgshA(pBBR::gshA)	2.5 µM Au, 10 µM Cu	2.90	2.32
Δcop Δcup Δgig	-	2.78	3.38
Δcop Δcup Δgig	2.5 µM Au	2.97	3.02
Δcop Δcup Δgig	2.5 µM Au, 10 µM Cu	2.92	2.43
Δcop Δcup Δgig ΔgshA	2.5 µM Au	2.34	2.83
Δcop Δcup Δgig ΔgshA(pBBR)	-	2.41	2.58
Δcop Δcup Δgig ΔgshA(pBBR)	2.5 µM Au	2.55	3.22
Δcop Δcup Δgig ΔgshA(pBBR)	2.5 µM Au, 10 µM Cu	2.47	3.15
Δcop Δcup Δgig ΔgshA(pBBR::gshA)	-	**3.76**	5.44
Δcop Δcup Δgig ΔgshA(pBBR::gshA)	2.5 µM Au	**4.37**	4.87
Δcop Δcup Δgig ΔgshA(pBBR::gshA)	2.5 µM Au, 10 µM Cu	**3.50**	3.01
Δcop Δcus Δgig	-	2.42	3.15
Δcop Δcus Δgig	2.5 µM Au	2.57	1.73
Δcop Δcus Δgig	2.5 µM Au, 10 µM Cu	2.21	1.96
Δcop Δcus Δgig ΔgshA(pBBR)	-	**3.17**	2.51
Δcop Δcus Δgig ΔgshA(pBBR)	2.5 µM Au	**3.10**	2.91
Δcop Δcus Δgig ΔgshA(pBBR)	2.5 µM Au, 10 µM Cu	2.92	4.54
Δcop Δcus Δgig ΔgshA(pBBR::gshA)	-	**3.26**	3.09
Δcop Δcus Δgig ΔgshA(pBBR::gshA)	2.5 µM Au	**3.87**	6.62
Δcop Δcus Δgig ΔgshA(pBBR::gshA)	2.5 µM Au, 10 µM Cu	**3.09**	1.88
Δcup Δcus Δgig	-	2.07	2.68
Δcup Δcus Δgig	2.5 µM Au	2.47	2.35
Δcup Δcus Δgig	2.5 µM Au, 10 µM Cu	2.27	1.70
Δcup Δcus Δgig ΔgshA(pBBR)	-	2.18	2.84
Δcup Δcus Δgig ΔgshA(pBBR)	2.5 µM Au	2.02	2.44
Δcup Δcus Δgig ΔgshA(pBBR::gshA)	-	2.55	2.33
Δcup Δcus Δgig ΔgshA(pBBR::gshA)	2.5 µM Au	2.88	3.35
Δcup Δcus Δgig ΔgshA(pBBR::gshA)	2.5 µM Au, 10 µM Cu	2.62	1.82

[a]This table presents from 780 ICP-MS measurements over all used strains and conditions the Mg contents that were more than 2-fold higher (Q > 2 and D > 1, mean values per strain and condition for all respective biological repeats) than the result from parent AE104 (no additions), 11.8±3.4 million atoms per cell. Exception is the *Δcop Δcup Δcus* value in the first line (in italics). The grey fields were used to cluster the same strain cultivated under different conditions. Values > 3 in bold-faced letters. Some data were already used to compose Suppl. Table S4 of a preceding publication (1).

Supplementary Table S3. Bacterial strains with cellular magnesium contents different from strain AE104 parent[a]

Bacterial Strain	Q(AE104)	D(AE104)
Δcop Δcus Δgig ΔgshA	0.49	1.27
Δcop Δcup Δgig ΔgshA	0.45	1.31
Δcop Δcup Δcus ΔgshA(pBBR)	0.48	1.41
Δcop Δcup Δcus Δgig ΔgshA(pBBR)	0.49	1.32

[a]This table presents from 780 ICP-MS measurements over all used strains and conditions the Fe contents that were more than 2-fold higher ($Q < 0.5$ and $D > 1$) than the result from parent AE104 (no additions), 787±265 thousand atoms per cell. All the listed strains were cultivated in the presence of 2.5 µM Au and 10 µM Cu. Some data were already used to compose Suppl. Table S4 of a preceding publication (1).

Supplementary Table S4. Bacterial strains

Bacterial strain	Description/genotype/plasmid	Reference
C. metallidurans		
AE104	Plasmid-free derivative of CH34	(2)
DN861	AE104 Δ*gshA*	(1)
DN656	AE104 Δ*cupCAR*	(3)
DN657	AE104 Δ*gigPABT*	(3)
DN686	AE104 Δ$copA_2B_2C_2D_2$	(3)
DN729	AE104 Δ*cusDCBAF*	(1)
DN845	AE104 Δ*cop* Δ*cup*	(1)
DN846	AE104 Δ*cop* Δ*cus*	(1)
DN847	AE104 Δ*cop* Δ*gig*	(1)
DN848	AE104 Δ*gig* Δ*cus*	(1)
DN849	AE104 Δ*cup* Δ*gig*	(1)
DN850	AE104 Δ*cup* Δ*cus*	(1)
DN851	AE104 Δ*gig* Δ*cus* Δ*cup*	(1)
DN852	AE104 Δgig Δ*cus* Δ*cop*	(1)
DN853	AE104 Δ*gig* Δ*cup* Δ*cop*	(1)
DN854	AE104 Δ*cop* Δ*cup* Δ*cus*	(1)
DN855	AE104 Δ*cop* Δ*cup* Δ*cus* Δ*gig*	(1)
DN994	no plasmid, Δ$copS_2$	this study
DN995	no plasmid, Δ$copR_2$	this study
DNA3	no plasmid, Δ$copR_2S_2$	this study
DN686	no plasmid, Δ$copD_2C_2B_2A_2$	(3)
E. coli (used for conjugation)		
ECA962	pECD1386, used for *cusF*-*lacZ* fusion	(1)
ECB249	pECD1667, used for *gigT*-*lacZ* fusion	(1)
ECB250	pECD1668, used for *gshA* disruption	(1)

Supplementary Table S5. Primers

Name and purpose	Sequence (5'→3')	Reference
Deletion of *gshA*		
RmgshAcreloxBglII	AAAAGATCTTGCCTGCGGGGCATAGAG	(1)
delta gshA-RM OR	CAGCAATTGGATGAGATGCGGGACCATATCCAT	(1)
Rm2 DgshA ApaI fwd	CTCGGGCCCACCGACCCGAATCCGATCCTC	(1)
Rm2 DgshA SacI rev	ATGGAGCTCGGCGGATCCTTGCGCATCA	(1)
Disruption of *gshA*		
gshA Rm dis 586 Pst	AAACTGCAGTACCTGCTGCCGCCGTTG	(1)
gshA Rm dis 901 Xba	AAATCTAGAGCCCTTCACCTCGGACGG	(1)
Deletion of *cusDCBAF*		
NW cus-like MunI	AAACAATTGGTGGATATCGGATCCAGAAC	(1)
NW cus-like NotI	AAAGCGGCCGCGATCTACGAGGATGTCAAAAC	(1)
NW cus-like ApaI	AAAGGGCCCCGCGATCGAGCCGCGCCGAC	(1)
NW cus-like AgeI	AAAACCGGTAGTGGGCCATCGCAATCGAAGGG	(1)
copR₂S₂ mutations		
*copS₂ Mun*I	AAA caa ttg CCT CGC TCG CCT TCG TGA	
*copS₂ Age*I	AAA acc ggt CGG GCT TGG CGT GCT GA	
copS₂ ApaI	AAA ggg ccc CGG TGT CCA TCC CCA GCA	
copS₂ NotI	AAA gcggccgc AAC GCG CAA GCC TGA TCA	
*copR₂ Mun*I	AAA caa ttg ACG ACA ATC CCC CGA GCG	
*copR₂ Age*I	AAA acc ggt GGT CCA TCG CCG TGA AGA	
*copR₂ Not*I	AAA gcggccgc CGT GTC CTC GTC AAA CCA ATG	
copR₂ ApaI	AAA ggg ccc CGC TGC TGG AAC TGC TGG	
*copR₂S₂ Mun*I	AAA caa ttg CAG TGC CAT TGC CCG TTG	
*copR₂S₂ Age*I	AAA acc ggt GAC CGT TTT CGT CAC CCA TCA	
*copR₂S₂ Not*I	AAA gcggccgc GGA AAA CGC CGC AAG TGT A	
copR₂S₂ ApaI	GCG ggg ccc GGG CAG AAG CCC GCC GTC	

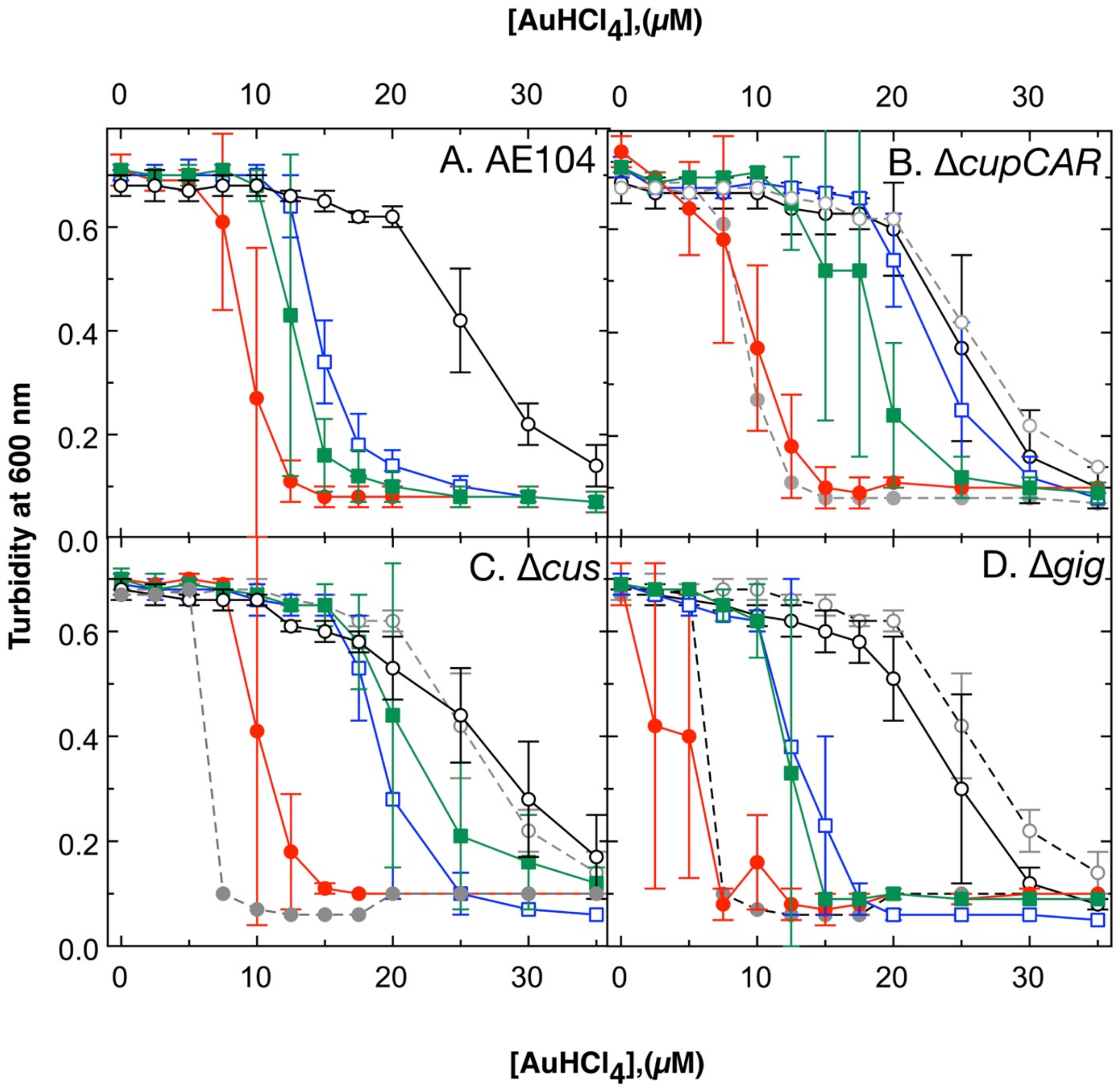

Supplementary Figure S1. Gold resistance of *C. metallidurans* mutant strains. Cells of the plasmid-free strain AE104 (Panel A, grey symbols and dashed lines in all other panels), its Δ*cupCAR* (Panel B), Δ*cus* (Panel C) or Δ*gig* (Panel D) derivate were cultivated with increasing gold concentrations ($Au(III)Cl_4^-$, previous gold solution) in the presence (filled symbols, ●, ■) or absence (open symbols, ❍, □) of 10 µM (A, B) or 100 µM (C, D) $CuCl_2$ in a 96 well plate in Tris-buffered mineral salts medium for 20 h with shaking at 30°C. The pre-cultures were incubated with 10 µM (A, B) or 100 µM (C, D) $CuCl_2$ (squares, □, ■) or not (circles, ❍, ●). The Optical Density (O.D.) at 600 nm was determined in a TECAN multiple plate reader. Deviations shown, n = 3. The results in Panel A are published (4) and shown for reference.

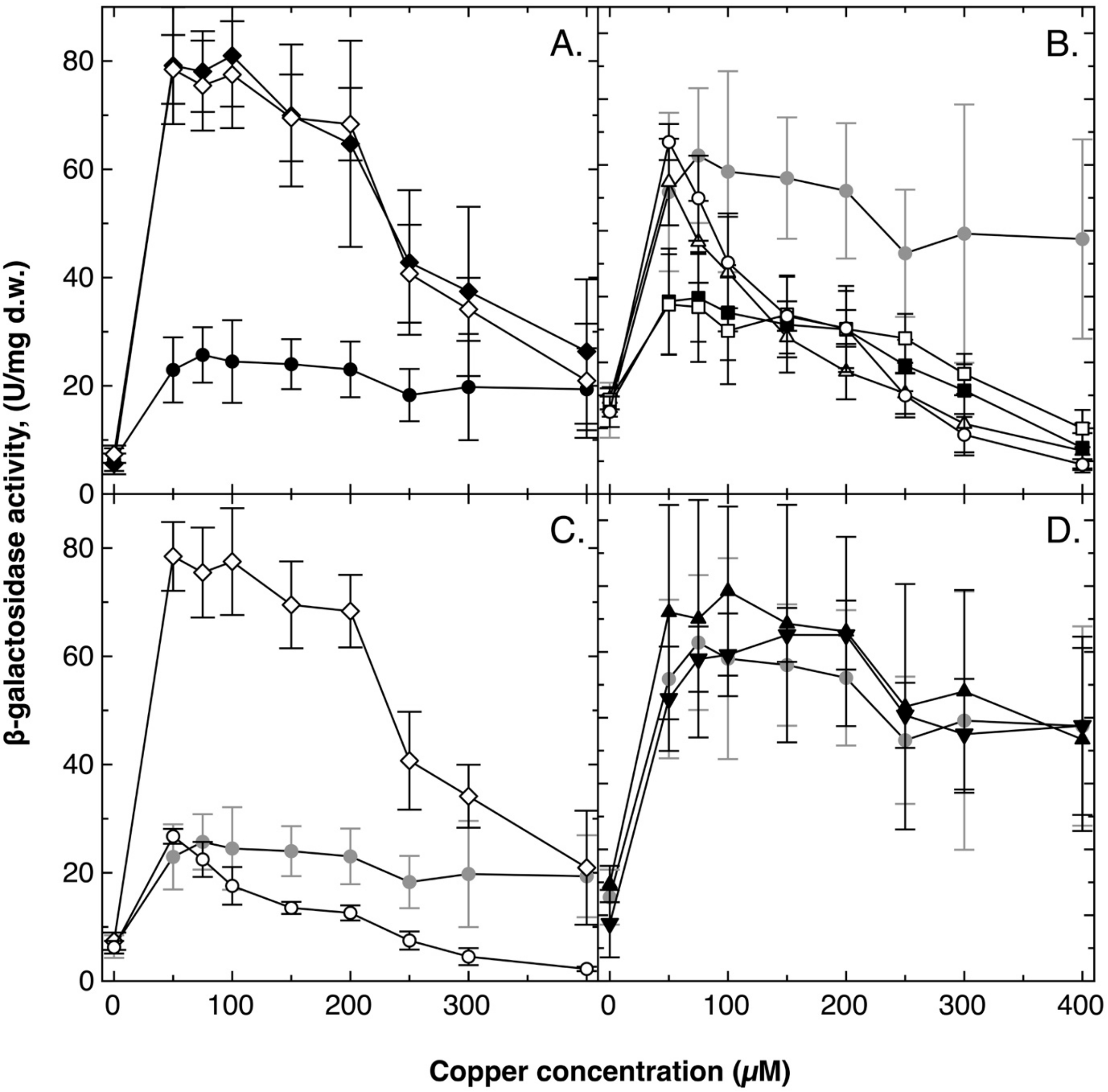

Supplementary Figure S2. Regulation of *cus* by copper ions. This published experiment (1) is shown for comparison. Reporter gene fusions with the *lacZ* gene were constructed with the *cus* operon in various mutant backgrounds. The strains were incubated in the presence of increasing copper concentrations and the beta-galactosidase activity was determined. In all panels: strain AE104 (●), black in Panel A, grey in all other panels for comparison.

Panel A: Δ*cop* (◆) and Δ*cop* Δ*gig* (◇).

Panel B: Δ*cup* (■), Δ*cup* Δ*gig* (□), Δ*cop* Δ*cup* (△) and Δ*cop* Δ*cup* Δ*gig* (○)

Panel C compares Δ*cop* Δ*gig* (◇) with Δ*cop* Δ*cup* Δ*gig* (○).

Panel D: Δ*gshA* (▼) and Δ*gig* (▲).

Deviations shown (n ≥ 3).

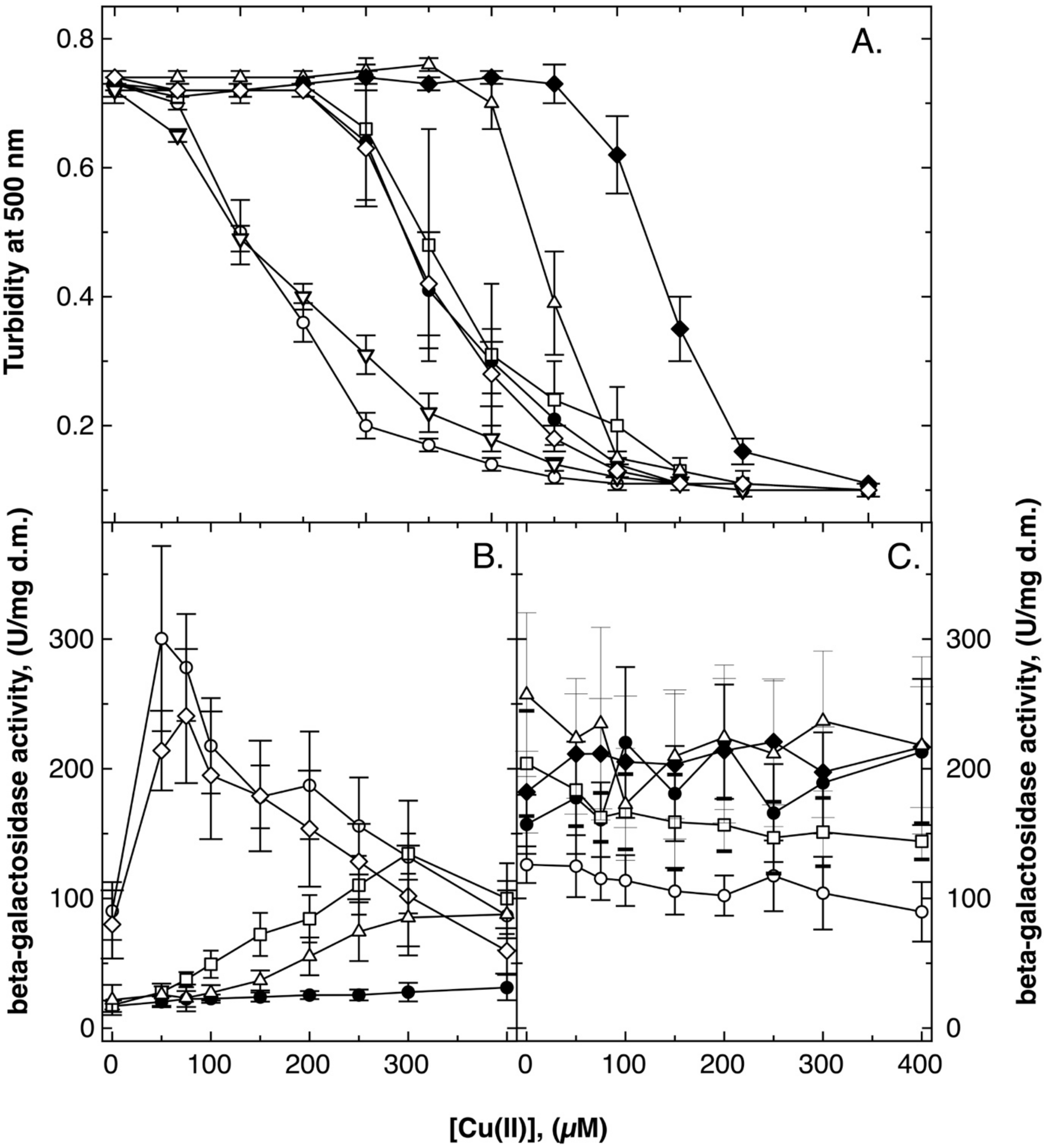

Supplementary Figure S3. Influence of Δ*copR₂S₂* deletions on copper resistance, expression of the *cus* and *cop₂* determinants. Panel A. Cells were cultivated in parallel cultures with increasing copper concentrations and growth was measured as turbidity at 600 nm after 24 h of incubation; n > 4. Panel B. Beta-galactosidase activity of a *cusF-lacZ* transcriptional fusion at various copper concentrations, n > 5. Panel C. Beta-galactosidase activity of a $copD_2$-*lacZ* transcriptional fusion at various copper concentrations, n > 7. Shown are *C. metallidurans* AE104 (●), Δ$copS_2$ (○), Δ$copR_2$ (□), Δ$copR_2S_2$ (Δ) in Panels B and C. The open diamonds (◇) represents Δ*copDCBA* in Panel B and Δ*cusDCBAF* in Panel C. Deviation bars are shown.

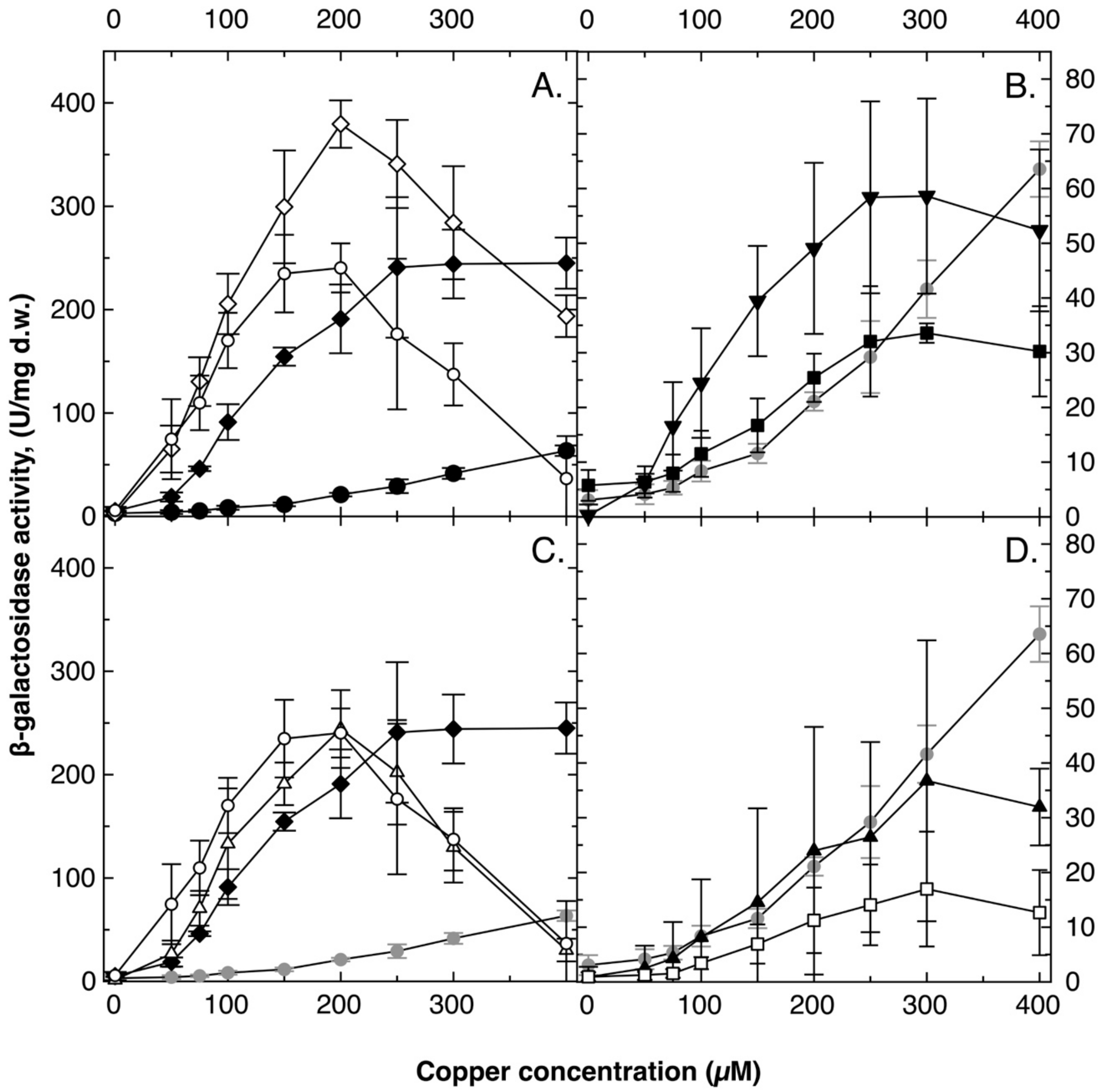

Supplementary Figure S4. Regulation of *gig* by copper ions. This published experiment (1) is shown for comparison. Reporter gene fusions with the *lacZ* gene were constructed with the *gig* operon in various mutant backgrounds. The strains were incubated in the presence of increasing copper concentrations and the beta-galactosidase activity was determined. In all panels: strain AE104 (●), black and enlarged in Panel A, grey in the other panels for comparison.

Panel A: Δ*cop* (◆), Δ*cop* Δ*cus* (◇), Δ*cop* Δ*cus* Δ*cup* (○).

Panel B: Δ*gshA* (▼) and Δ*cup* (■).

Panel C: Δ*cop* (◆) and Δ*cop* Δ*cus* Δ*cup* (○) in comparison with Δ*cop* Δ*cup* (△).

Panel D: Δ*cus* (▲) and Δ*cup* Δ*cus* (□).

Deviations shown (n ≥ 3).

Literature

1. Hirth N, Gerlach MS, Wiesemann N, Herzberg M, Grosse C, Nies DH. 2023. Full copper resistance in *Cupriavidus metallidurans* requires the interplay of many resistance systems. Appl Environ Microbiol 89:10.1128/aem.00567-23.
2. Mergeay M, Nies D, Schlegel HG, Gerits J, Charles P, van Gijsegem F. 1985. *Alcaligenes eutrophus* CH34 is a facultative chemolithotroph with plasmid-bound resistance to heavy metals. J Bacteriol 162:328-334.
3. Wiesemann N, Mohr J, Grosse C, Herzberg M, Hause G, Reith F, Nies DH. 2013. Influence of copper resistance determinants on gold transformation by *Cupriavidus metallidurans* strain CH34. J Bacteriol 195:2298-2308.
4. Wiesemann N, Bütof L, Herzberg M, Hause G, Berthold L, Etschmann B, Brugger J, Martinéz-Criado G, Dobritzsch D, Baginski S, Reith F, Nies DH. 2017. Synergistic toxicity of copper and gold compounds in *Cupriavidus metallidurans* Appl Environ Microbiol 83:e01679-17.
